# Dataset on the EEG time-frequency representation in children with different levels of mathematical achievement

**DOI:** 10.1016/j.dib.2018.10.105

**Published:** 2018-10-30

**Authors:** Andrés A. González-Garrido, Fabiola R. Gómez-Velázquez, Rebeca Romo-Vázquez, Hugo Vélez-Pérez, Ricardo A. Salido-Ruiz, Aurora Espinoza-Valdez, Geisa B. Gallardo-Moreno, Vanessa D. Ruiz-Stovel, Alicia Martínez-Ramos

**Affiliations:** a Instituto de Neurociencias, Universidad de Guadalajara, Francisco de Quevedo #180, Col. Arcos Vallarta, 44130 Guadalajara, Jalisco, México; b O.P.D. Hospital Civil de Guadalajara, Coronel Calderón #777, Col. El Retiro, CP. 44280 Guadalajara, Jalisco, México; c Departamento de Ciencias Computacionales, CUCEI, Universidad de Guadalajara, México; d Departamento de Neurociencias, CUCS, Universidad de Guadalajara, México

## Abstract

This article presents the data related to the research paper entitled “The analysis of EEG coherence reflects middle childhood differences in mathematical achievement” (González-Garrido et al., 2018). The dataset is derived from the electroencephalographic (EEG) records registered from a total of 60 8–9-years-old children with different math skill levels (High: HA, Average: AA, and Low Achievement: LA) while performing a symbolic magnitude comparison task. The average brain patterns are shown through Time-Frequency Representations (TFR) for each group, and also grand-mean amplitudes within specific EEG epochs in a 19-electrode array are provided. Making this information publicly available for further analyses could significantly contribute to a better understanding on how math achievement in children associates with cognitive processing strategies.

**Specifications table**

**Table:** 

Subject area	*Neuropsychology*
More specific subject area	*Numerical processing in children with different math skills*
Type of data	*Tables (Raw EEG data matrices), Figures.*
How data was acquired	*EEG was recorded from 19 scalp electrodes while performing a symbolic magnitude comparison task;*
	*Time windows corresponding to correct responses were selected;*
	*Time-Frequency was calculated for each group*
Data format	*Raw, Analyzed*
Experimental factors	*Three groups of children with different mathematical skills were instructed to perform a numerical comparison task with simultaneous EEG recording. The EEG characteristics were compared between the groups using a Surface Laplacian solution through a realistic head geometry model.*
Experimental features	*Time-Frequency Representation (TFR) was used to compare the EEG features among the groups.*
Data source location	*Instituto de Neurociencias, Universidad de Guadalajara, México.*
Data accessibility	*Data is with this article*
Related research article	González-Garrido, A.A., Gómez-Velázquez, F.R., Salido-Ruiz, R.A., Espinoza-Valdez, A., Vélez-Pérez, H., Romo-Vazquez, R., Gallardo-Moreno, G.B., Ruiz-Stovel, V.D., Martínez-Ramos, A., Berumen, G. (2018). The analysis of EEG coherence reflects middle childhood differences in mathematical achievement. Brain and Cognition, 124, 57-63 [1].

**Value of the data**

• The present data offers a significant contribution in the field of numerical processing in children. Data shows the brain functional relationship between math achievement and cognitive processing of numerical magnitudes.
• This data will be beneficial to understand how math achievement level in children associates with cognitive processing strategies.
• This data can be used to perform several signal processing analyses in order to evaluate different cognitive processes.

## 1 Data

**Fig. 1:**
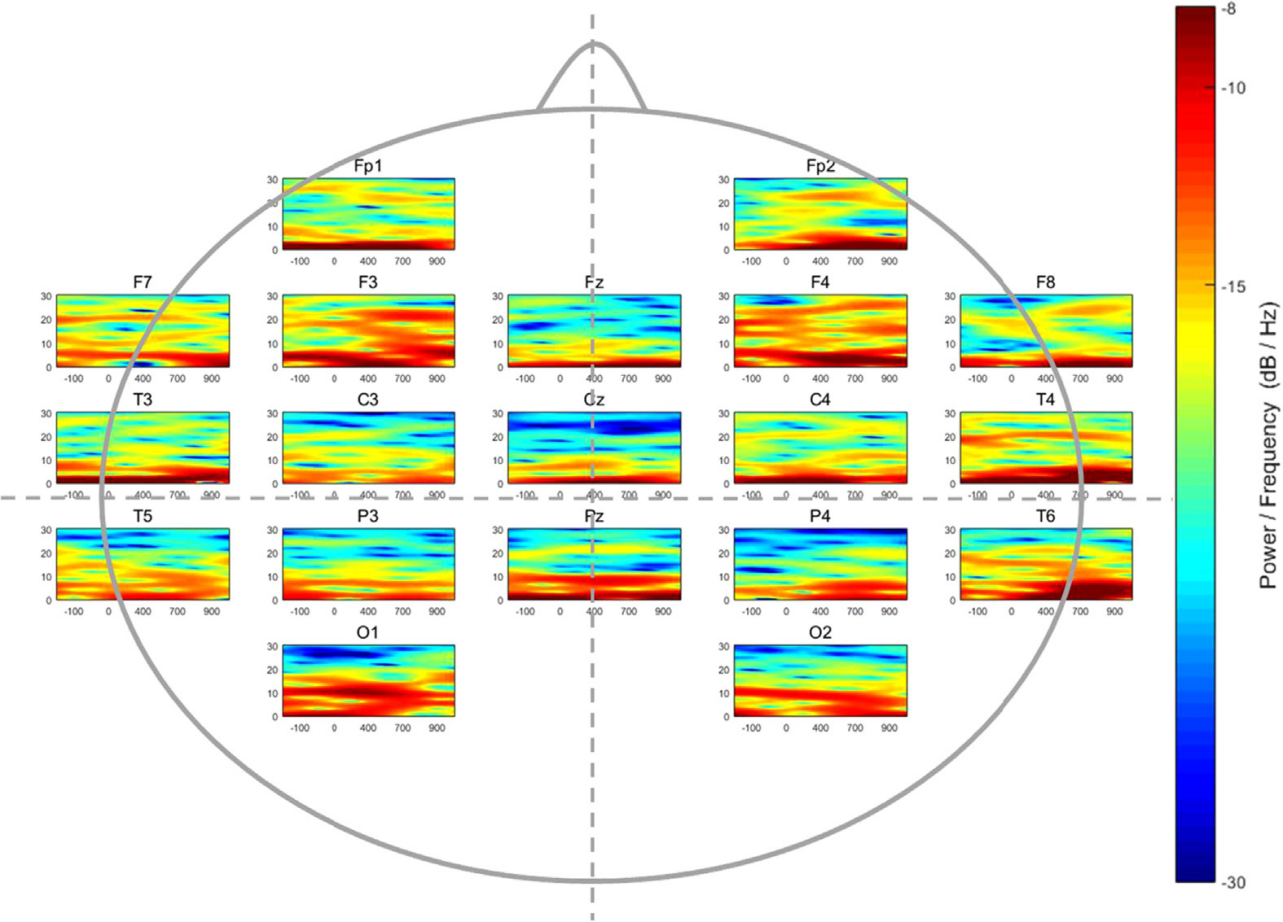
Time-Frequency distribution of the spectral power (dB/Hz) of 19 electrodes in the high mathematical achievement group while performing a symbolic magnitude comparison task.

**Fig. 2:**
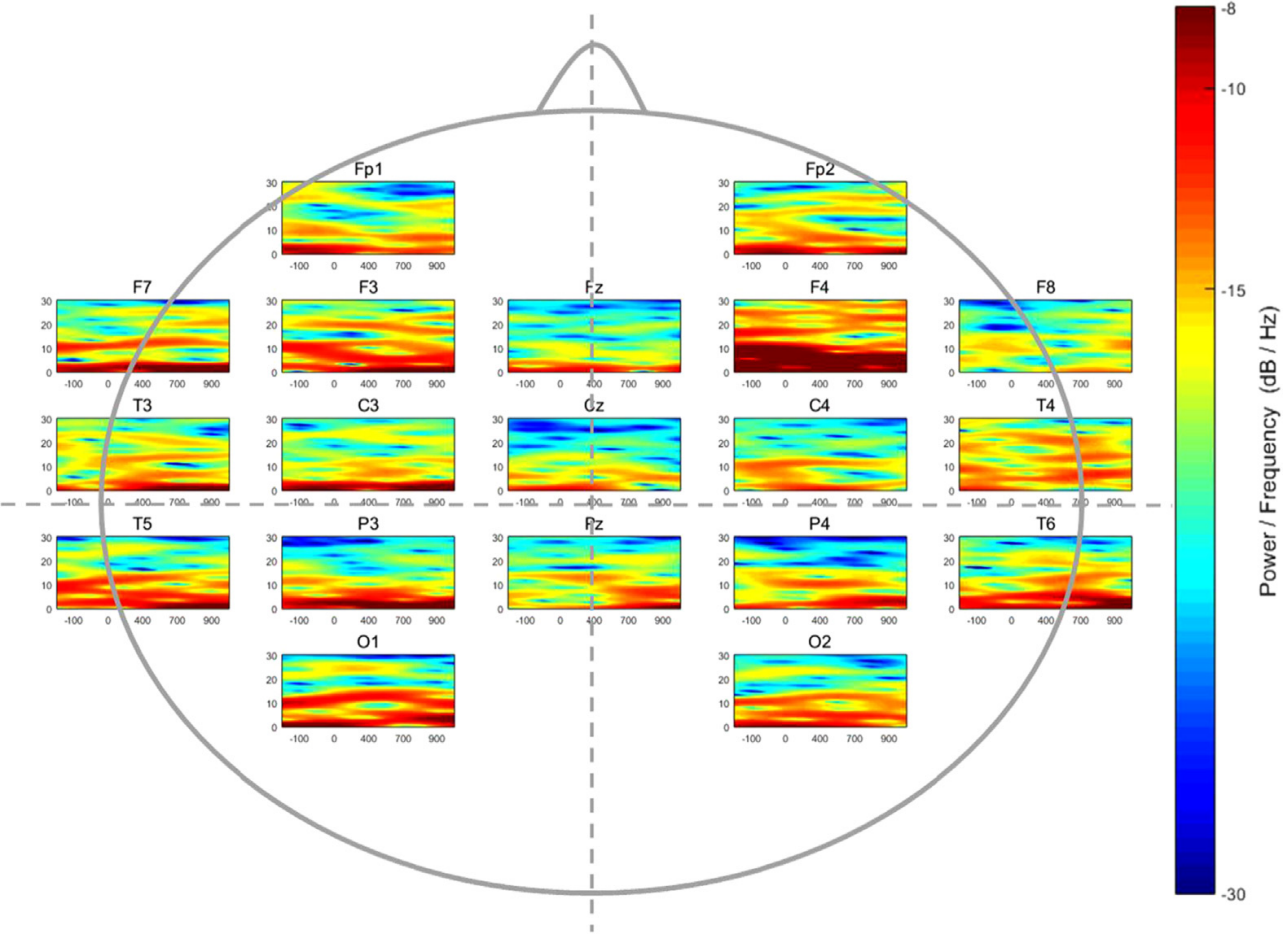
Time-Frequency distribution of the spectral power (dB/Hz) of 19 electrodes in the average mathematical achievement group while performing a symbolic magnitude comparison task.

**Fig. 3:**
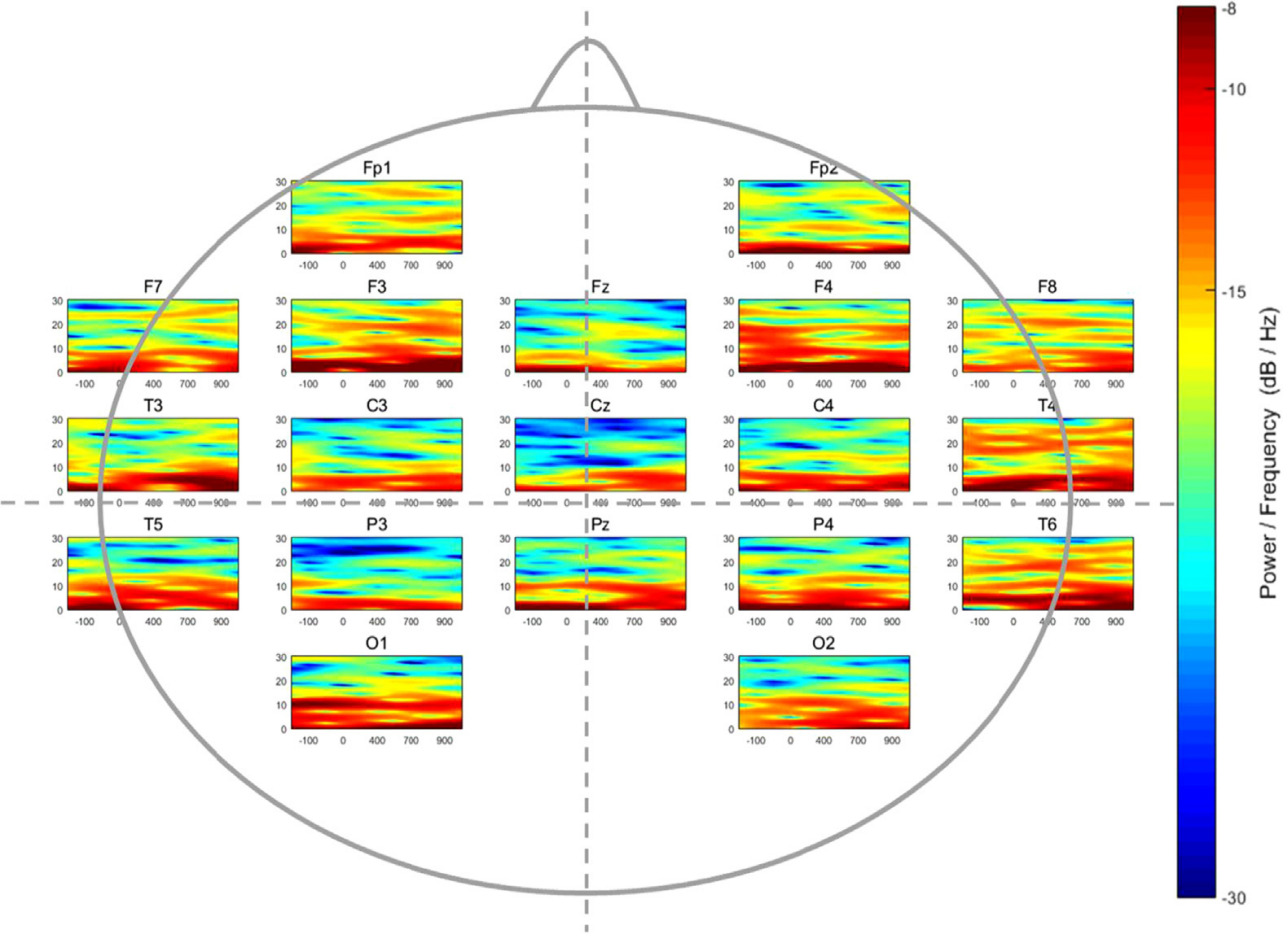
Time-Frequency distribution of the spectral power (dB/Hz) of 19 electrodes in the low mathematical achievement group while performing a symbolic magnitude comparison task.

The dataset presented provides information of the EEG from three groups of children with different math skill levels (High: HA, Average: AA, and Low achievement: LA) according to their scores in the Wide Range Achievement Test, 4th edition (WRAT-4) [2], while performing a symbolic magnitude comparison task (i.e., determining which of two numbers is numerically larger). Data collection was done with the aim of evaluating whether different levels of arithmetic skills in children might associate with distinct brain EEG patterns as a result of the acquired level of processing knowledge. In Tables 1–3, the dataset is organized in subsets, one per skill-level group, showing grand-mean amplitudes during EEG time-windows with correct responses in 19 electrode recording sites. In addition, Time-Frequency Representations (TFR) were calculated for each group, and the corresponding brain patterns are shown in Fig. 1, Fig. 2, Fig. 3.

## 2 Experimental design, materials, and methods

The EEG recordings were collected from a sample of 60 elementary school children between the ages of 8 and 9. These participants were selected from a pool of 441 third-graders from six public and three private elementary schools. They were equally divided into three groups of 20 according to their performance on the math sub-section of the WRAT-4: Low Achievement (LA; mean age = 9.1, SD = 0.62; 10 females) having scores within the lower 15th percentile; Average Achievement (AA; mean age = 9.1, SD = 0.48; 8 females) between the 30th and the 70th percentile; and High Achievement (HA; mean age = 9.1, SD = 0.33; 6 females) scoring in the upper 15th percentile. Children were matched according to their educational environment, such that each LA subject was paired with one AA and one HA children from the same school.

The experimental design is thoroughly described in González-Garrido et al. [1]. EEG activity was recorded from 19 scalp electrode sites, according to the International 10/20 system. From the EEG raw data, we selected individual time windows lasting 1000 ms, from 100 ms before to 900 ms after stimulus onset, corresponding only to correct behavioral responses. We selected 20 EEG epochs of 1000 ms duration from each participant. Grand-averaged voltage values (μV) in each recording site for the selected epochs appear in Tables 1–3. There is a corresponding table for each math level achievement group.

Afterwards, a Blind Source Separation method was used for artifact and noise canceling [3]. Later, a 3-layer realistic geometry head model of volume conduction was built in order to implement the Surface Laplacian solution through a realistic head geometry model [4], and a spatial filter was applied to all the EEG recordings prior to time-frequency estimation. In this dataset, the time-frequency coefficients were calculated among the 19 scalp locations for each participant. Later, the absolute power by channel was calculated and averaged in every single group. Fig. 1, Fig. 2, Fig. 3 show the averaged patterns of the EEG Time-Frequency Representations in HA, AA, and LA groups, respectively.

## Funding

Universidad de Guadalajara.
