# Supplementary material for: Dataset on the EEG time-frequency representation in children with different levels of mathematical achievement
Source: Data Brief. 2018 Oct 30;21:1071–5. doi: 10.1016/j.dib.2018.10.105 (PMC6226595; doi:10.1016/j.dib.2018.10.105)
Supplement: Supplementary file 2 — Supplementary material [file mmc2.docx]

**Table 1. Grand-mean voltages from EEG epochs with correct responses in children with high mathematical achievement level (n=20).**

| **Fp2** | **F4** | **C4** | **P4** | **O2** | **F8** | **T4** | **T6** | **Fz** | **Cz** | **Pz** | **Fp1** | **F3** | **C3** | **P3** | **O1** | **F7** | **T3** | **T5** |
| --- | --- | --- | --- | --- | --- | --- | --- | --- | --- | --- | --- | --- | --- | --- | --- | --- | --- | --- |
| 16.65 | 12.76 | 2.66 | 2.79 | 3.84 | 11.51 | 4.49 | 6.37 | 9.29 | 8.59 | 6.23 | 14.98 | 8.61 | 1.44 | 1.91 | 5.43 | 6.91 | 5.31 | 9.65 |
| 18.15 | 11.34 | 2.25 | 1.92 | 3.08 | 11.17 | 5.55 | 5.75 | 9.34 | 8.27 | 4.52 | 16.20 | 9.34 | 1.12 | 0.76 | 3.62 | 7.61 | 5.87 | 8.97 |
| 18.79 | 9.55 | 2.34 | 1.21 | 2.46 | 10.77 | 7.31 | 5.33 | 9.04 | 7.74 | 3.02 | 16.78 | 10.04 | 1.14 | -0.05 | 2.19 | 8.20 | 6.47 | 7.93 |
| 18.62 | 8.22 | 2.97 | 0.86 | 2.27 | 10.93 | 8.79 | 5.48 | 8.82 | 7.24 | 2.21 | 17.08 | 10.65 | 1.62 | -0.33 | 1.51 | 8.88 | 6.54 | 6.69 |
| 18.41 | 7.98 | 4.08 | 0.95 | 2.59 | 11.97 | 9.35 | 6.16 | 9.00 | 6.96 | 2.28 | 17.63 | 11.08 | 2.65 | 0.12 | 1.67 | 9.72 | 6.15 | 5.65 |
| 18.76 | 8.93 | 5.47 | 1.60 | 3.29 | 13.57 | 9.23 | 7.22 | 9.79 | 7.08 | 2.96 | 18.69 | 11.29 | 4.02 | 1.11 | 2.43 | 10.50 | 5.86 | 5.27 |
| 19.66 | 10.48 | 6.68 | 2.42 | 3.78 | 14.90 | 9.04 | 8.10 | 10.94 | 7.48 | 3.60 | 19.82 | 11.31 | 5.37 | 2.13 | 3.02 | 10.89 | 6.17 | 5.66 |
| 20.84 | 11.96 | 7.27 | 2.93 | 3.75 | 15.45 | 9.09 | 8.30 | 12.14 | 8.06 | 3.89 | 20.48 | 11.37 | 6.26 | 2.76 | 2.85 | 10.76 | 7.13 | 6.38 |
| 22.02 | 12.93 | 7.31 | 3.02 | 3.49 | 15.29 | 9.42 | 7.87 | 13.26 | 8.87 | 4.08 | 20.45 | 11.71 | 6.68 | 2.85 | 2.02 | 10.39 | 8.18 | 6.94 |
| 22.61 | 13.22 | 7.06 | 2.79 | 3.05 | 14.81 | 9.75 | 6.84 | 14.03 | 9.83 | 4.56 | 19.62 | 12.20 | 6.96 | 2.66 | 1.09 | 10.21 | 8.63 | 6.99 |
| 22.13 | 13.13 | 6.93 | 2.39 | 2.33 | 14.34 | 9.63 | 5.40 | 14.40 | 10.80 | 5.24 | 18.77 | 12.59 | 7.36 | 2.31 | 0.47 | 10.70 | 8.50 | 6.37 |
| 21.01 | 12.86 | 7.08 | 1.99 | 1.28 | 14.04 | 8.92 | 3.88 | 14.48 | 11.55 | 5.84 | 18.69 | 12.64 | 8.00 | 1.96 | 0.27 | 11.70 | 8.08 | 5.51 |
| 20.39 | 12.43 | 7.45 | 1.84 | 0.26 | 14.07 | 7.94 | 2.65 | 14.36 | 11.96 | 6.16 | 19.28 | 12.50 | 8.72 | 1.75 | 0.34 | 12.49 | 7.56 | 4.89 |
| 20.81 | 11.77 | 7.75 | 1.93 | -0.51 | 14.50 | 7.50 | 1.93 | 14.05 | 11.97 | 6.40 | 20.00 | 12.41 | 9.12 | 1.78 | 0.52 | 12.56 | 6.88 | 4.71 |
| 21.51 | 10.90 | 7.64 | 2.18 | -1.02 | 15.05 | 8.15 | 1.45 | 13.30 | 11.42 | 6.67 | 20.09 | 12.36 | 8.81 | 2.03 | 0.71 | 12.00 | 6.05 | 4.87 |
| 21.37 | 10.27 | 7.13 | 2.51 | -1.30 | 15.17 | 9.32 | 1.19 | 12.09 | 10.51 | 7.20 | 19.52 | 11.94 | 7.78 | 2.57 | 1.08 | 11.24 | 5.34 | 5.20 |
| 20.09 | 10.24 | 6.60 | 3.11 | -1.21 | 14.75 | 9.98 | 1.61 | 10.64 | 9.57 | 7.98 | 18.23 | 10.99 | 6.43 | 3.37 | 1.75 | 10.34 | 4.79 | 5.64 |
| 18.26 | 10.58 | 6.31 | 4.07 | -0.75 | 14.05 | 9.73 | 2.90 | 9.44 | 8.85 | 8.92 | 16.16 | 9.80 | 5.33 | 4.32 | 2.62 | 9.13 | 4.30 | 6.02 |
| 16.64 | 10.47 | 6.18 | 5.16 | -0.18 | 13.55 | 9.08 | 4.71 | 8.73 | 8.40 | 9.52 | 13.85 | 8.75 | 4.73 | 5.16 | 3.28 | 7.83 | 3.75 | 6.02 |
| 15.49 | 9.19 | 6.02 | 5.96 | 0.35 | 13.40 | 8.75 | 6.35 | 8.46 | 8.09 | 9.58 | 12.23 | 8.08 | 4.46 | 5.71 | 3.54 | 6.90 | 3.28 | 5.75 |
| 14.95 | 7.16 | 5.75 | 6.45 | 1.09 | 13.26 | 8.73 | 7.58 | 8.64 | 7.90 | 9.32 | 12.05 | 7.94 | 4.43 | 6.09 | 3.53 | 6.63 | 2.99 | 5.53 |
| 15.13 | 5.33 | 5.44 | 6.69 | 2.26 | 12.89 | 8.51 | 8.78 | 8.99 | 7.86 | 9.07 | 13.14 | 8.13 | 4.42 | 6.42 | 3.50 | 7.05 | 2.74 | 5.64 |
| 15.84 | 4.46 | 5.17 | 6.79 | 3.73 | 12.10 | 7.56 | 10.06 | 9.25 | 7.69 | 8.96 | 14.38 | 8.26 | 4.31 | 6.75 | 3.68 | 7.71 | 2.62 | 6.16 |
| 16.29 | 4.36 | 5.07 | 7.11 | 5.20 | 11.05 | 6.56 | 11.31 | 9.07 | 7.32 | 9.02 | 14.88 | 7.98 | 4.11 | 7.07 | 4.44 | 8.31 | 2.85 | 7.18 |
| 15.77 | 4.29 | 5.29 | 7.71 | 6.43 | 9.89 | 6.72 | 12.08 | 8.36 | 6.90 | 9.28 | 14.63 | 7.29 | 3.87 | 7.40 | 5.90 | 8.71 | 3.53 | 8.74 |
| 14.57 | 4.14 | 5.79 | 8.55 | 7.45 | 8.85 | 7.98 | 12.32 | 7.55 | 6.50 | 9.48 | 14.05 | 6.57 | 3.53 | 7.61 | 7.66 | 9.02 | 4.04 | 10.22 |
| 13.60 | 3.88 | 6.27 | 9.31 | 8.26 | 8.27 | 9.06 | 12.40 | 7.15 | 6.20 | 9.43 | 13.55 | 6.14 | 3.08 | 7.53 | 8.93 | 9.29 | 3.82 | 11.07 |
| 13.48 | 3.82 | 6.44 | 9.64 | 8.79 | 8.34 | 9.03 | 12.75 | 7.38 | 5.85 | 8.91 | 13.08 | 6.21 | 2.51 | 7.11 | 9.34 | 9.40 | 3.17 | 11.13 |
| 13.85 | 4.14 | 6.16 | 9.42 | 8.82 | 9.01 | 8.04 | 13.37 | 8.12 | 5.52 | 8.05 | 12.33 | 6.71 | 1.97 | 6.38 | 9.16 | 9.30 | 2.68 | 10.61 |
| 14.15 | 4.59 | 5.63 | 8.73 | 8.19 | 9.94 | 7.01 | 13.73 | 9.10 | 5.30 | 7.26 | 11.53 | 7.33 | 1.61 | 5.59 | 8.76 | 9.02 | 2.44 | 9.69 |
| 14.32 | 4.72 | 5.09 | 7.72 | 6.77 | 10.52 | 6.30 | 13.33 | 9.95 | 5.38 | 6.87 | 11.23 | 7.59 | 1.51 | 4.90 | 8.23 | 8.54 | 2.13 | 8.38 |
| 14.49 | 4.31 | 4.77 | 6.70 | 4.86 | 10.47 | 5.84 | 12.22 | 10.57 | 5.56 | 6.78 | 11.66 | 7.05 | 1.68 | 4.45 | 7.60 | 7.84 | 1.67 | 7.12 |
| 14.80 | 3.51 | 4.65 | 5.85 | 2.84 | 9.92 | 5.80 | 10.72 | 10.85 | 5.62 | 6.61 | 12.68 | 5.92 | 2.04 | 3.98 | 6.78 | 7.01 | 1.68 | 6.34 |
| 14.93 | 2.77 | 4.43 | 5.00 | 0.94 | 9.21 | 6.08 | 8.91 | 10.59 | 5.15 | 5.90 | 13.53 | 4.54 | 2.26 | 3.24 | 5.66 | 6.46 | 2.62 | 5.85 |
| 14.54 | 2.35 | 3.88 | 3.77 | -0.94 | 8.49 | 6.25 | 6.60 | 9.69 | 4.17 | 4.65 | 13.53 | 3.28 | 2.13 | 2.17 | 4.33 | 6.58 | 3.89 | 5.28 |
| 13.77 | 2.36 | 3.19 | 2.23 | -2.54 | 8.24 | 6.34 | 4.11 | 8.46 | 3.03 | 3.37 | 12.67 | 2.54 | 1.74 | 1.23 | 3.22 | 7.31 | 4.85 | 4.46 |
| 13.11 | 2.78 | 2.88 | 1.01 | -3.45 | 8.83 | 6.55 | 2.14 | 7.52 | 2.36 | 2.65 | 11.44 | 2.66 | 1.50 | 0.91 | 2.87 | 8.35 | 5.19 | 3.59 |
| 12.88 | 3.64 | 3.21 | 0.53 | -3.47 | 10.09 | 6.71 | 1.21 | 7.31 | 2.44 | 2.62 | 10.54 | 3.53 | 1.50 | 1.11 | 3.09 | 9.25 | 5.08 | 2.81 |
| 13.17 | 4.52 | 4.08 | 0.76 | -2.70 | 11.05 | 6.31 | 1.40 | 7.75 | 3.14 | 2.91 | 10.59 | 4.55 | 1.65 | 1.34 | 3.17 | 9.88 | 4.68 | 2.05 |
| 13.79 | 4.93 | 5.19 | 1.43 | -1.44 | 11.00 | 5.50 | 2.24 | 8.40 | 4.14 | 3.36 | 11.96 | 5.06 | 1.69 | 1.35 | 2.83 | 10.12 | 4.02 | 1.44 |
| 14.47 | 4.61 | 6.21 | 2.38 | -0.15 | 9.94 | 5.33 | 3.41 | 8.81 | 5.22 | 4.10 | 14.06 | 4.99 | 1.65 | 1.15 | 2.30 | 9.93 | 3.43 | 1.20 |
| 14.96 | 3.78 | 6.73 | 3.49 | 0.64 | 8.59 | 6.04 | 4.58 | 8.77 | 6.07 | 5.06 | 15.42 | 4.59 | 1.58 | 0.90 | 1.80 | 9.35 | 3.22 | 1.35 |
| 15.11 | 2.88 | 6.70 | 4.57 | 1.00 | 7.82 | 6.93 | 5.42 | 8.28 | 6.55 | 6.08 | 15.29 | 4.27 | 1.51 | 0.63 | 1.37 | 8.50 | 3.34 | 1.67 |
| 15.04 | 2.30 | 6.32 | 5.36 | 1.38 | 7.86 | 7.14 | 5.70 | 7.47 | 6.70 | 6.92 | 14.16 | 4.06 | 1.32 | 0.21 | 0.81 | 7.60 | 3.15 | 1.57 |
| 14.74 | 2.18 | 5.95 | 5.76 | 2.17 | 8.19 | 6.69 | 5.43 | 6.49 | 6.73 | 7.40 | 13.02 | 3.83 | 0.88 | -0.39 | 0.03 | 6.87 | 2.27 | 0.92 |
| 14.16 | 2.45 | 5.84 | 5.78 | 3.35 | 8.26 | 6.52 | 4.97 | 5.57 | 6.82 | 7.55 | 12.34 | 3.40 | 0.20 | -1.19 | -0.84 | 6.47 | 1.05 | 0.11 |
| 13.06 | 2.88 | 5.90 | 5.67 | 4.55 | 8.06 | 7.21 | 4.86 | 4.86 | 7.12 | 7.56 | 11.64 | 2.87 | -0.50 | -2.00 | -1.60 | 6.30 | 0.12 | -0.40 |
| 11.59 | 3.23 | 6.04 | 5.62 | 5.44 | 8.07 | 8.22 | 5.32 | 4.34 | 7.56 | 7.63 | 10.58 | 2.32 | -0.94 | -2.56 | -1.90 | 6.14 | -0.25 | -0.75 |
| 10.40 | 3.02 | 6.06 | 5.62 | 5.75 | 8.46 | 8.86 | 6.01 | 3.93 | 7.83 | 7.81 | 9.38 | 1.96 | -1.03 | -2.76 | -1.74 | 5.84 | -0.26 | -1.22 |
| 10.13 | 2.17 | 6.01 | 5.65 | 5.29 | 8.66 | 8.83 | 6.33 | 3.49 | 7.76 | 8.05 | 8.60 | 1.77 | -0.90 | -2.72 | -1.40 | 5.40 | -0.11 | -1.86 |
| 10.55 | 0.90 | 5.95 | 5.57 | 4.20 | 8.08 | 7.99 | 6.05 | 3.04 | 7.42 | 8.34 | 8.63 | 1.83 | -0.71 | -2.60 | -1.29 | 5.27 | -0.07 | -2.40 |
| 11.05 | 0.01 | 5.96 | 5.35 | 2.74 | 7.18 | 6.65 | 5.43 | 2.85 | 6.94 | 8.48 | 9.18 | 2.20 | -0.53 | -2.43 | -1.33 | 5.77 | -0.20 | -2.54 |
| 10.99 | 0.10 | 6.00 | 4.89 | 1.30 | 6.75 | 5.37 | 4.79 | 2.96 | 6.36 | 8.34 | 9.33 | 2.75 | -0.35 | -2.15 | -1.17 | 6.63 | -0.60 | -2.23 |
| 10.47 | 0.92 | 6.05 | 4.37 | 0.51 | 7.28 | 4.70 | 4.45 | 3.36 | 5.61 | 7.96 | 8.73 | 3.41 | -0.28 | -1.78 | -0.43 | 7.31 | -0.94 | -1.62 |
| 10.04 | 1.63 | 6.12 | 3.98 | 0.73 | 8.24 | 4.94 | 4.49 | 3.83 | 4.75 | 7.42 | 7.88 | 3.93 | -0.38 | -1.31 | 0.65 | 7.61 | -1.04 | -0.80 |
| 9.98 | 1.63 | 6.15 | 3.69 | 1.59 | 8.81 | 5.66 | 4.51 | 4.26 | 3.87 | 6.78 | 7.68 | 4.20 | -0.75 | -0.97 | 1.56 | 7.75 | -0.80 | 0.03 |
| 10.07 | 1.25 | 5.77 | 3.14 | 2.14 | 8.64 | 6.58 | 3.97 | 4.45 | 2.87 | 5.74 | 8.18 | 4.08 | -1.50 | -1.03 | 1.85 | 7.99 | -0.59 | 0.60 |
| 10.30 | 1.17 | 4.83 | 2.14 | 2.00 | 8.08 | 7.77 | 2.83 | 4.35 | 1.64 | 4.22 | 8.73 | 3.52 | -2.72 | -1.76 | 1.49 | 8.24 | -0.67 | 0.76 |
| 10.79 | 1.54 | 3.67 | 1.01 | 1.62 | 7.86 | 8.58 | 1.67 | 4.05 | 0.38 | 2.43 | 9.10 | 2.77 | -4.09 | -2.85 | 0.96 | 8.46 | -0.99 | 0.73 |
| 11.53 | 1.89 | 3.01 | 0.26 | 1.79 | 8.29 | 8.30 | 1.29 | 3.86 | -0.60 | 1.03 | 9.32 | 2.14 | -5.22 | -3.79 | 0.73 | 8.49 | -1.28 | 0.61 |
| 12.12 | 1.83 | 3.19 | 0.39 | 2.65 | 9.22 | 7.12 | 1.97 | 3.97 | -1.10 | 0.53 | 9.43 | 1.95 | -5.74 | -4.19 | 0.84 | 8.32 | -1.23 | 0.52 |
| 12.08 | 1.45 | 3.98 | 1.15 | 3.89 | 10.20 | 5.83 | 3.22 | 4.16 | -1.26 | 0.81 | 9.52 | 2.21 | -5.75 | -4.02 | 0.93 | 8.08 | -0.93 | 0.31 |
| 11.59 | 1.10 | 4.43 | 1.93 | 4.85 | 10.73 | 4.83 | 4.28 | 3.96 | -1.51 | 1.32 | 9.67 | 2.52 | -5.61 | -3.60 | 0.66 | 7.76 | -0.69 | -0.04 |
| 11.33 | 1.00 | 4.04 | 2.19 | 5.22 | 10.54 | 4.01 | 4.53 | 3.23 | -1.89 | 1.63 | 9.96 | 2.43 | -5.32 | -3.10 | 0.35 | 7.21 | -0.77 | -0.40 |
| 11.77 | 1.19 | 3.00 | 1.93 | 5.15 | 9.96 | 3.25 | 4.04 | 2.38 | -2.23 | 1.75 | 10.39 | 1.84 | -4.88 | -2.59 | 0.25 | 6.47 | -0.82 | -0.46 |
| 12.85 | 1.84 | 2.27 | 1.76 | 4.93 | 9.52 | 2.89 | 3.50 | 2.15 | -2.03 | 2.08 | 11.18 | 1.40 | -4.11 | -2.03 | 0.31 | 6.19 | -0.22 | -0.02 |
| 14.11 | 2.81 | 2.42 | 2.16 | 4.77 | 9.48 | 3.22 | 3.55 | 2.94 | -1.15 | 2.79 | 12.34 | 1.82 | -3.05 | -1.47 | 0.33 | 6.94 | 1.19 | 0.77 |
| 14.71 | 3.60 | 3.28 | 3.10 | 4.70 | 9.68 | 3.79 | 4.05 | 4.36 | 0.19 | 3.76 | 13.13 | 3.16 | -1.73 | -0.79 | 0.47 | 8.47 | 2.96 | 1.68 |
| 13.98 | 3.84 | 4.34 | 4.37 | 4.76 | 9.86 | 4.48 | 4.35 | 5.73 | 1.69 | 4.79 | 12.50 | 4.80 | -0.25 | 0.14 | 1.10 | 9.82 | 4.20 | 2.63 |
| 12.06 | 3.38 | 5.19 | 5.62 | 4.97 | 9.93 | 5.60 | 4.14 | 6.66 | 3.10 | 5.84 | 10.45 | 6.03 | 1.30 | 1.39 | 2.24 | 10.23 | 4.63 | 3.82 |
| 10.24 | 2.84 | 5.84 | 6.50 | 5.26 | 10.07 | 6.86 | 3.70 | 7.08 | 4.31 | 6.90 | 8.52 | 6.64 | 2.80 | 2.79 | 3.59 | 10.03 | 4.86 | 5.27 |
| 9.81 | 2.61 | 6.24 | 6.73 | 5.31 | 10.01 | 7.42 | 3.33 | 7.13 | 5.36 | 7.77 | 8.35 | 6.67 | 3.99 | 3.93 | 4.67 | 9.81 | 5.64 | 6.60 |
| 11.00 | 2.91 | 6.50 | 6.48 | 5.08 | 9.87 | 6.67 | 3.07 | 7.12 | 6.21 | 8.49 | 10.17 | 6.41 | 4.79 | 4.65 | 5.36 | 9.93 | 6.98 | 7.45 |
| 13.01 | 3.78 | 6.85 | 6.22 | 4.75 | 10.13 | 5.07 | 2.88 | 7.55 | 7.22 | 9.25 | 12.92 | 6.51 | 5.38 | 5.11 | 6.00 | 10.42 | 8.15 | 7.96 |
| 14.66 | 5.06 | 7.48 | 6.25 | 4.48 | 10.84 | 3.59 | 2.73 | 8.70 | 8.29 | 9.93 | 14.93 | 7.39 | 5.86 | 5.31 | 6.69 | 10.97 | 8.50 | 8.18 |
| 14.84 | 6.27 | 8.12 | 6.34 | 4.22 | 11.45 | 2.97 | 2.65 | 10.25 | 9.09 | 10.15 | 15.08 | 8.96 | 6.09 | 5.10 | 7.03 | 11.38 | 7.75 | 7.87 |
| 13.32 | 6.92 | 8.51 | 6.31 | 3.99 | 11.57 | 3.37 | 2.89 | 11.43 | 9.30 | 9.70 | 13.44 | 10.49 | 5.94 | 4.22 | 6.82 | 11.42 | 6.45 | 6.86 |
| 11.02 | 6.81 | 8.71 | 6.57 | 3.85 | 11.24 | 4.27 | 3.56 | 11.99 | 9.10 | 8.83 | 11.36 | 11.57 | 5.52 | 3.25 | 6.36 | 11.35 | 5.34 | 5.68 |
| 9.53 | 6.24 | 8.92 | 7.36 | 3.97 | 10.91 | 4.97 | 4.66 | 12.16 | 8.91 | 8.10 | 10.32 | 12.02 | 5.26 | 2.69 | 6.23 | 11.35 | 5.10 | 5.09 |
| 9.57 | 5.50 | 9.18 | 8.33 | 4.37 | 10.65 | 5.05 | 5.89 | 12.04 | 8.74 | 7.66 | 10.80 | 11.92 | 5.21 | 2.65 | 6.35 | 11.68 | 5.75 | 5.30 |
| 10.71 | 4.69 | 9.15 | 8.76 | 4.97 | 9.93 | 4.76 | 6.80 | 11.40 | 8.24 | 7.18 | 12.20 | 10.98 | 5.04 | 2.58 | 5.95 | 12.02 | 6.52 | 5.83 |
| 11.99 | 3.73 | 8.73 | 8.39 | 5.58 | 8.79 | 4.37 | 7.21 | 10.17 | 7.36 | 6.42 | 13.27 | 9.56 | 4.59 | 2.13 | 4.50 | 11.87 | 6.62 | 5.92 |
| 12.76 | 2.99 | 8.25 | 7.71 | 6.14 | 7.86 | 3.85 | 7.48 | 9.05 | 6.56 | 5.51 | 13.22 | 8.60 | 4.24 | 1.44 | 2.30 | 11.07 | 5.81 | 5.41 |
| 12.96 | 3.00 | 7.99 | 7.59 | 6.60 | 7.83 | 3.13 | 7.95 | 8.75 | 6.28 | 4.87 | 12.17 | 8.85 | 4.45 | 1.06 | 0.30 | 10.17 | 4.82 | 4.69 |
| 12.71 | 3.78 | 8.05 | 8.10 | 6.86 | 8.92 | 2.84 | 8.48 | 9.38 | 6.51 | 4.79 | 10.98 | 10.07 | 5.19 | 1.39 | -0.90 | 9.86 | 4.35 | 4.40 |
| 12.13 | 4.75 | 8.25 | 8.76 | 6.97 | 10.69 | 3.44 | 8.83 | 10.35 | 6.80 | 5.23 | 10.60 | 11.40 | 6.14 | 2.33 | -1.10 | 10.28 | 4.28 | 4.78 |
| 11.27 | 5.13 | 8.51 | 9.18 | 7.19 | 12.00 | 4.60 | 8.91 | 10.96 | 6.94 | 5.88 | 11.06 | 12.07 | 6.95 | 3.57 | -0.55 | 10.77 | 4.20 | 5.52 |
| 10.07 | 4.86 | 8.86 | 9.36 | 7.47 | 12.08 | 5.75 | 8.73 | 10.84 | 6.70 | 6.47 | 11.49 | 11.96 | 7.41 | 4.80 | 0.42 | 10.73 | 3.97 | 6.05 |
| 8.63 | 4.21 | 9.20 | 9.52 | 7.79 | 10.92 | 6.35 | 8.54 | 10.00 | 6.01 | 6.98 | 10.89 | 11.38 | 7.37 | 5.86 | 1.92 | 10.04 | 3.93 | 6.35 |
| 7.35 | 3.50 | 9.47 | 9.76 | 8.00 | 9.43 | 6.12 | 8.54 | 8.76 | 4.91 | 7.48 | 9.38 | 10.58 | 7.01 | 6.88 | 3.94 | 9.10 | 4.50 | 6.77 |
| 6.30 | 2.94 | 9.54 | 10.06 | 8.01 | 8.39 | 5.11 | 8.73 | 7.41 | 3.69 | 8.08 | 7.54 | 9.55 | 6.60 | 7.79 | 5.99 | 8.31 | 5.62 | 7.65 |
| 5.33 | 2.44 | 9.32 | 10.34 | 7.85 | 7.62 | 4.19 | 8.91 | 5.89 | 2.70 | 8.68 | 5.95 | 8.12 | 6.45 | 8.49 | 7.52 | 7.87 | 6.71 | 8.78 |
| 4.46 | 2.00 | 8.82 | 10.57 | 7.54 | 6.65 | 4.35 | 8.93 | 4.32 | 2.28 | 9.28 | 4.73 | 6.57 | 6.58 | 8.87 | 8.34 | 7.71 | 7.25 | 9.55 |
| 4.28 | 1.86 | 8.32 | 10.70 | 6.96 | 5.56 | 5.40 | 8.70 | 3.03 | 2.35 | 9.80 | 3.95 | 5.45 | 6.97 | 9.01 | 8.62 | 7.64 | 7.10 | 9.58 |
| 5.11 | 2.03 | 8.02 | 10.60 | 6.07 | 5.08 | 6.47 | 8.29 | 2.48 | 2.84 | 10.16 | 3.84 | 5.17 | 7.38 | 9.05 | 8.69 | 7.61 | 6.60 | 9.01 |
| 6.37 | 2.47 | 8.10 | 10.13 | 4.86 | 5.79 | 7.09 | 7.68 | 2.88 | 3.61 | 10.30 | 4.72 | 5.61 | 7.55 | 8.91 | 8.48 | 7.77 | 6.23 | 8.44 |
| 7.41 | 3.21 | 8.60 | 9.35 | 3.81 | 7.60 | 7.52 | 6.73 | 3.94 | 4.60 | 10.14 | 6.20 | 6.45 | 7.42 | 8.52 | 7.86 | 8.26 | 6.06 | 8.06 |
| 8.21 | 4.28 | 9.30 | 8.47 | 3.02 | 9.84 | 8.08 | 5.57 | 5.18 | 5.82 | 9.84 | 7.47 | 7.38 | 7.25 | 7.74 | 6.69 | 8.96 | 5.82 | 7.63 |
| 9.04 | 5.70 | 9.79 | 7.60 | 2.24 | 11.72 | 8.61 | 4.55 | 6.26 | 6.96 | 9.36 | 8.04 | 8.30 | 7.08 | 6.48 | 4.93 | 9.57 | 5.11 | 6.64 |
| 9.79 | 6.80 | 9.56 | 6.70 | 1.01 | 12.52 | 8.33 | 4.04 | 6.94 | 7.57 | 8.42 | 8.07 | 8.98 | 6.59 | 4.62 | 2.51 | 9.84 | 3.83 | 5.09 |
| 10.03 | 7.00 | 8.62 | 5.88 | -0.38 | 12.15 | 7.23 | 4.31 | 7.21 | 7.42 | 6.91 | 8.11 | 9.33 | 5.65 | 2.45 | -0.27 | 9.71 | 2.78 | 3.44 |
| 9.80 | 6.27 | 7.49 | 5.44 | -0.92 | 11.20 | 5.76 | 5.32 | 7.20 | 6.66 | 5.23 | 8.30 | 9.38 | 4.66 | 0.70 | -2.64 | 9.13 | 2.42 | 2.07 |
| 9.39 | 5.25 | 6.57 | 5.30 | -0.15 | 10.13 | 4.51 | 6.69 | 7.05 | 5.64 | 3.79 | 8.38 | 9.30 | 3.98 | -0.25 | -4.02 | 8.21 | 2.77 | 1.13 |
| 9.03 | 4.49 | 5.74 | 5.17 | 1.40 | 9.18 | 3.88 | 7.89 | 6.98 | 4.53 | 2.63 | 8.11 | 9.13 | 3.70 | -0.51 | -4.40 | 7.26 | 3.29 | 0.63 |
| 8.49 | 4.18 | 4.61 | 4.64 | 2.80 | 8.30 | 3.85 | 8.49 | 6.99 | 3.25 | 1.39 | 7.78 | 8.98 | 3.59 | -0.56 | -4.14 | 7.01 | 3.64 | 0.57 |
| 7.83 | 4.20 | 3.06 | 3.51 | 3.46 | 7.47 | 3.72 | 8.17 | 6.88 | 1.71 | -0.31 | 7.68 | 8.86 | 3.40 | -0.82 | -3.56 | 7.56 | 3.96 | 1.08 |
| 7.15 | 4.17 | 1.24 | 1.97 | 3.33 | 6.70 | 2.97 | 7.02 | 6.43 | -0.13 | -2.55 | 7.45 | 8.53 | 2.96 | -1.43 | -2.79 | 8.12 | 4.32 | 1.96 |
| 6.46 | 3.84 | -0.19 | 0.67 | 2.95 | 6.04 | 1.88 | 5.72 | 5.56 | -1.90 | -4.71 | 6.56 | 7.70 | 2.20 | -2.06 | -1.66 | 7.92 | 4.62 | 2.88 |
| 5.52 | 3.12 | -0.67 | 0.37 | 3.15 | 5.83 | 1.15 | 5.41 | 4.60 | -3.16 | -5.84 | 5.09 | 6.43 | 1.38 | -2.15 | 0.27 | 6.93 | 4.71 | 3.65 |
| 4.58 | 2.22 | 0.07 | 1.35 | 4.42 | 6.27 | 1.19 | 6.51 | 3.83 | -3.67 | -5.40 | 3.68 | 5.03 | 0.66 | -1.34 | 3.14 | 5.51 | 4.46 | 4.60 |
| 4.06 | 1.33 | 1.25 | 2.96 | 6.51 | 6.94 | 1.67 | 8.37 | 3.09 | -3.75 | -3.85 | 2.91 | 3.64 | 0.15 | 0.07 | 6.41 | 4.12 | 3.75 | 5.94 |
| 4.05 | 0.37 | 2.11 | 4.15 | 8.32 | 7.27 | 2.03 | 9.78 | 2.15 | -3.99 | -2.47 | 2.63 | 2.16 | -0.36 | 1.38 | 9.16 | 2.96 | 2.69 | 7.29 |
| 4.04 | -0.61 | 2.42 | 4.75 | 9.31 | 7.10 | 2.54 | 10.42 | 1.27 | -4.54 | -1.76 | 2.43 | 0.80 | -0.86 | 2.18 | 10.93 | 2.41 | 1.91 | 8.17 |
| 3.48 | -1.11 | 2.59 | 5.31 | 9.92 | 6.81 | 3.39 | 10.75 | 1.04 | -4.93 | -1.40 | 2.07 | -0.04 | -0.96 | 2.61 | 11.86 | 2.73 | 2.12 | 8.36 |
| 2.00 | -0.52 | 2.98 | 6.26 | 10.61 | 6.68 | 4.45 | 11.42 | 1.76 | -4.72 | -0.82 | 1.37 | -0.04 | -0.43 | 3.14 | 12.42 | 3.52 | 3.02 | 8.26 |
| 0.02 | 1.10 | 3.48 | 7.38 | 11.21 | 6.45 | 5.37 | 12.32 | 2.86 | -3.99 | 0.23 | 0.31 | 0.52 | 0.40 | 3.93 | 12.66 | 3.99 | 3.86 | 8.51 |
| -1.65 | 2.98 | 3.71 | 8.18 | 11.10 | 6.03 | 5.71 | 13.10 | 3.60 | -3.05 | 1.48 | -0.67 | 1.00 | 1.10 | 5.01 | 12.42 | 3.66 | 4.17 | 9.34 |
| -2.49 | 4.13 | 3.68 | 8.61 | 10.11 | 5.63 | 5.54 | 13.61 | 3.65 | -2.11 | 2.76 | -1.01 | 0.93 | 1.52 | 6.24 | 11.69 | 2.83 | 3.97 | 10.63 |
| -2.32 | 4.03 | 3.57 | 8.98 | 8.63 | 5.67 | 4.80 | 13.99 | 3.11 | -1.25 | 3.92 | -0.85 | 0.25 | 1.74 | 7.36 | 10.53 | 1.95 | 3.62 | 11.85 |
| -1.38 | 2.85 | 3.52 | 9.49 | 7.05 | 6.15 | 3.85 | 14.35 | 2.13 | -0.46 | 4.87 | -0.98 | -0.76 | 2.05 | 8.08 | 9.18 | 1.36 | 3.44 | 12.54 |
| -0.06 | 1.28 | 3.42 | 10.05 | 5.54 | 6.84 | 3.56 | 14.70 | 1.13 | 0.20 | 5.48 | -1.49 | -1.62 | 2.49 | 8.28 | 8.04 | 1.10 | 3.82 | 12.63 |
| 1.11 | 0.35 | 3.33 | 10.37 | 4.16 | 7.51 | 4.15 | 14.78 | 0.47 | 0.72 | 5.63 | -1.65 | -1.84 | 3.07 | 7.92 | 7.18 | 1.19 | 4.66 | 12.41 |
| 1.27 | 0.45 | 3.25 | 10.23 | 3.19 | 8.19 | 4.95 | 14.32 | 0.56 | 1.04 | 5.34 | -0.93 | -1.39 | 3.68 | 7.13 | 6.46 | 1.66 | 5.20 | 12.15 |
| 0.47 | 1.18 | 3.17 | 9.49 | 2.67 | 8.68 | 5.05 | 13.00 | 1.41 | 1.01 | 4.49 | 0.23 | -0.64 | 4.20 | 6.05 | 5.40 | 2.44 | 4.89 | 11.68 |
| -0.47 | 1.96 | 3.04 | 8.01 | 2.09 | 8.76 | 4.59 | 10.76 | 2.58 | 0.52 | 3.04 | 0.97 | 0.13 | 4.48 | 4.84 | 3.83 | 3.21 | 4.01 | 10.71 |
| -0.53 | 2.72 | 2.84 | 5.89 | 1.11 | 8.53 | 4.46 | 8.15 | 3.60 | -0.41 | 1.03 | 1.08 | 0.71 | 4.38 | 3.58 | 1.90 | 3.56 | 3.23 | 9.11 |
| 0.21 | 3.55 | 2.59 | 3.48 | -0.28 | 8.09 | 5.08 | 5.93 | 4.15 | -1.38 | -1.18 | 0.89 | 1.10 | 4.03 | 2.33 | 0.07 | 3.42 | 2.82 | 7.30 |
| 1.05 | 4.26 | 2.38 | 1.22 | -1.54 | 7.53 | 5.97 | 4.65 | 4.20 | -2.03 | -3.16 | 0.88 | 1.26 | 3.65 | 1.19 | -1.22 | 2.94 | 2.61 | 5.76 |
| 1.46 | 4.59 | 2.16 | -0.56 | -2.46 | 6.97 | 6.51 | 4.01 | 4.07 | -2.13 | -4.69 | 1.35 | 1.22 | 3.45 | 0.30 | -1.94 | 2.65 | 2.57 | 4.67 |
| 1.71 | 4.57 | 2.02 | -1.79 | -3.11 | 6.63 | 6.32 | 3.28 | 4.15 | -1.74 | -5.60 | 2.18 | 1.35 | 3.41 | -0.23 | -2.11 | 3.26 | 2.80 | 4.04 |
| 2.49 | 4.73 | 2.05 | -2.41 | -3.43 | 6.86 | 5.82 | 2.09 | 4.62 | -1.10 | -6.00 | 3.12 | 1.96 | 3.54 | -0.42 | -2.01 | 4.67 | 3.33 | 3.66 |
| 4.13 | 5.51 | 2.36 | -2.52 | -3.25 | 7.86 | 5.40 | 0.77 | 5.53 | -0.48 | -6.12 | 4.19 | 3.11 | 3.75 | -0.45 | -1.85 | 6.42 | 3.87 | 3.19 |
| 6.20 | 6.76 | 2.74 | -2.46 | -2.66 | 9.33 | 5.45 | -0.13 | 6.52 | 0.00 | -6.34 | 5.60 | 4.37 | 4.11 | -0.69 | -1.87 | 7.71 | 4.21 | 2.42 |
| 7.40 | 7.84 | 2.74 | -2.78 | -2.41 | 10.64 | 6.14 | -0.66 | 7.19 | 0.10 | -6.98 | 6.84 | 4.96 | 4.28 | -1.48 | -2.22 | 8.19 | 4.16 | 1.22 |
| 6.68 | 8.06 | 2.18 | -3.75 | -3.05 | 11.04 | 7.16 | -1.31 | 7.09 | -0.33 | -8.04 | 7.04 | 4.53 | 4.05 | -2.78 | -3.16 | 8.09 | 3.94 | -0.13 |
| 4.63 | 7.52 | 1.34 | -5.05 | -4.37 | 10.24 | 8.20 | -2.35 | 6.40 | -1.05 | -9.12 | 5.98 | 3.43 | 3.49 | -4.05 | -4.54 | 7.72 | 4.13 | -1.14 |
| 2.82 | 6.93 | 0.81 | -5.82 | -5.21 | 8.88 | 8.66 | -3.31 | 5.85 | -1.40 | -9.38 | 4.69 | 2.52 | 3.38 | -4.48 | -5.36 | 7.54 | 5.17 | -1.22 |
| 2.42 | 7.07 | 1.03 | -5.45 | -4.95 | 8.26 | 8.36 | -3.64 | 6.11 | -0.92 | -8.36 | 4.36 | 2.39 | 4.07 | -3.48 | -4.74 | 7.69 | 6.85 | -0.16 |
| 3.53 | 8.03 | 1.99 | -3.89 | -3.96 | 9.07 | 8.00 | -3.16 | 7.31 | 0.38 | -6.35 | 5.11 | 3.30 | 5.57 | -1.39 | -2.64 | 8.15 | 8.59 | 1.55 |
| 5.57 | 9.64 | 3.33 | -1.67 | -2.77 | 11.06 | 8.58 | -2.19 | 9.11 | 2.11 | -4.01 | 6.39 | 4.98 | 7.37 | 1.00 | -0.17 | 8.92 | 9.81 | 3.08 |
| 7.92 | 11.43 | 4.64 | 0.67 | -1.78 | 13.23 | 10.37 | -1.17 | 10.88 | 4.00 | -1.84 | 7.77 | 6.95 | 9.01 | 3.06 | 1.39 | 9.99 | 10.50 | 3.88 |
| 9.88 | 12.85 | 5.70 | 2.65 | -1.04 | 14.70 | 12.25 | -0.51 | 12.18 | 5.93 | 0.07 | 8.88 | 8.56 | 10.34 | 4.53 | 1.76 | 11.22 | 11.08 | 4.12 |
| 10.49 | 13.23 | 6.54 | 4.18 | -0.68 | 15.17 | 13.02 | -0.17 | 12.85 | 7.78 | 1.76 | 9.42 | 9.32 | 11.34 | 5.58 | 1.39 | 12.37 | 11.45 | 4.25 |
| 9.82 | 12.34 | 7.47 | 5.48 | -0.69 | 14.81 | 12.83 | -0.06 | 12.91 | 9.31 | 3.37 | 9.37 | 9.36 | 12.19 | 6.39 | 0.67 | 13.09 | 11.34 | 4.33 |
| 8.56 | 10.84 | 8.54 | 6.60 | -0.76 | 14.15 | 12.62 | 0.01 | 12.67 | 10.47 | 4.84 | 9.08 | 9.44 | 13.13 | 7.08 | -0.22 | 13.37 | 10.67 | 4.01 |
| 7.30 | 9.88 | 9.57 | 7.48 | -0.75 | 13.55 | 13.12 | 0.16 | 12.44 | 11.25 | 6.05 | 8.98 | 10.04 | 14.23 | 7.69 | -1.21 | 13.35 | 9.86 | 3.05 |
| 6.32 | 10.11 | 10.28 | 8.02 | -0.70 | 13.10 | 13.93 | 0.39 | 12.45 | 11.77 | 6.99 | 8.93 | 10.96 | 15.21 | 8.25 | -1.96 | 13.45 | 9.59 | 1.96 |
| 5.82 | 11.06 | 10.51 | 8.18 | -0.71 | 12.46 | 14.06 | 0.67 | 12.79 | 12.05 | 7.58 | 8.75 | 11.79 | 15.72 | 8.78 | -2.10 | 13.79 | 10.09 | 1.39 |
| 5.97 | 11.61 | 10.21 | 8.14 | -0.71 | 11.59 | 13.25 | 0.86 | 13.45 | 12.00 | 7.79 | 8.75 | 12.34 | 15.60 | 9.15 | -1.50 | 14.48 | 11.05 | 1.59 |
| 6.91 | 11.23 | 9.49 | 7.99 | -0.54 | 10.85 | 12.29 | 0.95 | 14.19 | 11.70 | 7.53 | 9.21 | 12.83 | 14.97 | 9.16 | -0.57 | 15.52 | 12.00 | 2.07 |
| 8.25 | 10.30 | 8.59 | 7.90 | -0.18 | 10.64 | 12.38 | 1.09 | 14.66 | 11.29 | 6.90 | 10.12 | 13.36 | 14.15 | 8.75 | 0.34 | 16.74 | 12.49 | 2.28 |
| 9.24 | 9.48 | 7.77 | 7.90 | 0.53 | 10.75 | 13.77 | 1.64 | 14.58 | 10.93 | 6.15 | 11.19 | 13.59 | 13.30 | 7.98 | 1.10 | 17.60 | 12.32 | 2.20 |
| 9.09 | 8.91 | 7.01 | 7.72 | 1.16 | 10.47 | 14.98 | 2.44 | 13.64 | 10.40 | 5.42 | 11.80 | 13.09 | 12.44 | 7.07 | 1.76 | 17.36 | 11.65 | 2.27 |
| 7.34 | 8.30 | 6.09 | 6.98 | 1.15 | 9.42 | 14.15 | 3.00 | 11.95 | 9.23 | 4.40 | 11.45 | 11.74 | 11.26 | 6.01 | 2.01 | 15.83 | 10.97 | 2.56 |
| 4.70 | 7.40 | 4.89 | 5.60 | 0.43 | 7.70 | 11.28 | 2.92 | 10.02 | 7.33 | 2.86 | 9.90 | 9.89 | 9.67 | 4.70 | 1.58 | 13.77 | 10.48 | 2.66 |
| 2.53 | 6.35 | 3.78 | 4.03 | -0.69 | 5.88 | 8.42 | 2.33 | 8.48 | 5.23 | 1.12 | 7.60 | 8.13 | 8.09 | 3.37 | 0.64 | 12.49 | 9.89 | 2.44 |
| 1.60 | 5.32 | 3.15 | 2.88 | -1.77 | 4.58 | 7.40 | 1.75 | 7.62 | 3.61 | -0.14 | 5.62 | 6.94 | 7.05 | 2.47 | -0.26 | 12.62 | 8.97 | 1.92 |
| 1.57 | 4.34 | 2.90 | 2.27 | -2.42 | 4.16 | 8.03 | 1.42 | 7.20 | 2.60 | -0.56 | 4.93 | 6.32 | 6.72 | 2.21 | -0.61 | 13.52 | 8.29 | 1.69 |
| 1.79 | 3.40 | 2.48 | 1.92 | -2.58 | 4.70 | 8.84 | 1.26 | 6.75 | 1.83 | -0.45 | 5.55 | 5.80 | 6.86 | 2.28 | -0.38 | 14.35 | 8.61 | 2.21 |
| 2.13 | 2.35 | 1.51 | 1.47 | -2.40 | 5.68 | 8.77 | 1.14 | 5.91 | 0.75 | -0.50 | 6.71 | 5.07 | 6.94 | 2.17 | -0.03 | 14.60 | 9.81 | 3.22 |
| 2.92 | 1.48 | 0.19 | 0.85 | -2.09 | 6.41 | 7.99 | 0.98 | 4.84 | -0.60 | -1.04 | 7.73 | 4.28 | 6.71 | 1.51 | 0.11 | 14.44 | 10.86 | 4.00 |
| 4.13 | 0.90 | -0.87 | 0.26 | -1.87 | 6.37 | 7.16 | 1.05 | 3.84 | -1.86 | -1.91 | 8.59 | 3.84 | 6.22 | 0.49 | 0.10 | 14.06 | 11.24 | 3.98 |
| 5.16 | 0.68 | -1.36 | -0.17 | -1.79 | 5.55 | 6.70 | 1.20 | 3.18 | -2.69 | -2.80 | 9.36 | 3.78 | 5.59 | -0.53 | -0.02 | 13.47 | 11.05 | 3.21 |
| 5.18 | 0.66 | -1.40 | -0.54 | -1.95 | 4.48 | 6.79 | 1.04 | 2.89 | -3.04 | -3.53 | 9.45 | 3.73 | 4.99 | -1.23 | -0.25 | 12.46 | 10.62 | 2.18 |
| 3.99 | 0.47 | -1.36 | -1.06 | -2.65 | 3.66 | 7.20 | 0.36 | 2.81 | -3.07 | -4.05 | 8.26 | 3.52 | 4.38 | -1.51 | -0.78 | 11.10 | 9.62 | 1.40 |
| 2.02 | -0.04 | -1.43 | -1.81 | -3.73 | 3.25 | 7.44 | -0.52 | 2.87 | -2.93 | -4.28 | 6.18 | 3.37 | 3.81 | -1.52 | -1.75 | 9.62 | 8.04 | 1.24 |
| 0.10 | -0.85 | -1.63 | -2.48 | -4.77 | 3.15 | 7.15 | -1.02 | 2.94 | -2.76 | -4.26 | 4.55 | 3.44 | 3.21 | -1.49 | -2.84 | 8.62 | 6.38 | 1.57 |
| -0.87 | -1.70 | -1.99 | -2.86 | -5.13 | 3.28 | 6.45 | -0.83 | 2.83 | -2.57 | -4.09 | 4.25 | 3.56 | 2.42 | -1.65 | -3.73 | 8.25 | 5.30 | 1.97 |
| -0.53 | -2.09 | -2.52 | -2.95 | -4.68 | 3.63 | 5.94 | -0.28 | 2.67 | -2.43 | -3.95 | 4.98 | 3.49 | 1.45 | -2.06 | -4.27 | 8.37 | 4.64 | 2.16 |
| 0.94 | -1.92 | -3.16 | -3.01 | -3.82 | 4.10 | 5.80 | 0.09 | 2.66 | -2.21 | -3.75 | 6.12 | 3.26 | 0.56 | -2.66 | -4.58 | 8.66 | 4.02 | 2.03 |
| 2.62 | -1.41 | -3.82 | -3.21 | -3.21 | 4.35 | 5.76 | -0.15 | 2.97 | -1.84 | -3.66 | 7.08 | 3.33 | 0.02 | -3.35 | -4.96 | 8.93 | 3.50 | 1.70 |
| 3.48 | -0.97 | -4.39 | -3.65 | -2.96 | 4.01 | 5.47 | -0.89 | 3.38 | -1.42 | -3.55 | 7.43 | 3.84 | -0.15 | -3.92 | -5.51 | 8.85 | 3.43 | 1.18 |
| 3.27 | -0.84 | -4.96 | -4.19 | -2.84 | 3.28 | 4.89 | -1.79 | 3.53 | -1.09 | -3.50 | 6.96 | 4.42 | -0.31 | -4.24 | -5.98 | 8.40 | 3.92 | 0.51 |
| 2.56 | -0.95 | -5.58 | -4.62 | -2.59 | 2.90 | 4.39 | -2.35 | 3.37 | -0.96 | -3.34 | 5.79 | 4.74 | -0.63 | -4.20 | -5.80 | 7.93 | 4.71 | -0.07 |
| 2.08 | -1.03 | -6.01 | -4.87 | -2.26 | 3.26 | 4.12 | -2.45 | 3.09 | -0.92 | -3.05 | 4.80 | 4.74 | -0.96 | -3.73 | -4.72 | 8.10 | 5.81 | -0.14 |
| 2.14 | -0.94 | -6.10 | -4.99 | -2.05 | 3.92 | 3.86 | -2.24 | 3.09 | -0.78 | -2.52 | 4.78 | 4.63 | -0.95 | -2.86 | -2.97 | 9.31 | 7.22 | 0.51 |
| 2.14 | -1.03 | -5.86 | -5.27 | -2.34 | 4.17 | 3.77 | -2.28 | 3.26 | -0.43 | -2.03 | 5.63 | 4.67 | -0.63 | -1.95 | -1.25 | 10.80 | 8.83 | 1.51 |
| 1.65 | -1.55 | -5.52 | -5.77 | -3.03 | 3.76 | 4.36 | -2.94 | 3.40 | -0.09 | -1.90 | 6.39 | 4.79 | -0.31 | -1.32 | -0.10 | 11.64 | 9.90 | 2.42 |
| 0.65 | -2.19 | -5.30 | -6.24 | -3.52 | 3.01 | 5.32 | -4.01 | 3.38 | 0.08 | -1.99 | 6.32 | 5.09 | -0.01 | -0.93 | 0.41 | 11.55 | 9.81 | 3.09 |
| -0.49 | -2.39 | -5.12 | -6.33 | -3.30 | 2.10 | 5.85 | -4.94 | 3.26 | 0.04 | -2.08 | 5.29 | 5.49 | 0.52 | -0.50 | 0.60 | 10.97 | 8.77 | 3.61 |
| -1.61 | -2.10 | -4.93 | -5.91 | -2.22 | 0.98 | 5.42 | -5.17 | 3.19 | -0.18 | -1.99 | 3.85 | 5.72 | 1.43 | 0.29 | 0.82 | 10.25 | 7.59 | 4.17 |
| -2.55 | -1.69 | -4.64 | -5.15 | -0.53 | 0.02 | 4.42 | -4.59 | 3.06 | -0.38 | -1.71 | 2.56 | 5.76 | 2.59 | 1.49 | 1.37 | 9.44 | 7.15 | 4.72 |
| -2.75 | -1.31 | -4.33 | -4.29 | 1.12 | -0.25 | 3.55 | -3.31 | 2.88 | -0.43 | -1.25 | 2.06 | 5.64 | 3.88 | 2.95 | 2.27 | 8.70 | 7.65 | 5.51 |
| -1.82 | -0.78 | -4.08 | -3.59 | 2.43 | 0.25 | 3.00 | -1.74 | 2.67 | -0.41 | -0.67 | 2.36 | 5.47 | 5.12 | 4.49 | 3.60 | 8.32 | 8.71 | 6.87 |
| -0.31 | -0.20 | -3.89 | -3.03 | 3.28 | 0.92 | 2.76 | -0.20 | 2.57 | -0.48 | 0.06 | 2.75 | 5.34 | 6.01 | 5.86 | 5.24 | 8.36 | 9.61 | 8.66 |
| 0.76 | -0.32 | -3.77 | -2.58 | 3.64 | 1.17 | 3.05 | 1.21 | 2.66 | -0.62 | 0.74 | 2.49 | 5.34 | 6.22 | 6.66 | 7.04 | 8.28 | 9.99 | 10.09 |
| 0.74 | -1.68 | -3.60 | -2.11 | 3.55 | 1.01 | 3.77 | 2.59 | 2.89 | -0.78 | 1.29 | 1.46 | 5.35 | 5.64 | 6.77 | 8.49 | 7.75 | 9.84 | 10.42 |
| -0.11 | -3.89 | -3.20 | -1.37 | 3.29 | 0.62 | 4.56 | 4.04 | 3.23 | -0.82 | 1.71 | 0.30 | 5.09 | 4.71 | 6.37 | 9.06 | 6.73 | 9.33 | 9.69 |
| -1.49 | -5.77 | -2.63 | -0.43 | 3.02 | 0.07 | 5.11 | 5.33 | 3.46 | -0.75 | 2.13 | -0.53 | 4.24 | 3.75 | 5.81 | 8.48 | 5.33 | 8.59 | 8.45 |
| -2.95 | -6.44 | -2.09 | 0.53 | 2.96 | -0.56 | 5.47 | 6.04 | 3.41 | -0.60 | 2.53 | -0.76 | 2.93 | 2.90 | 5.30 | 6.97 | 3.89 | 7.57 | 7.19 |
| -3.91 | -6.01 | -1.64 | 1.27 | 3.29 | -1.05 | 5.69 | 6.11 | 2.89 | -0.41 | 2.95 | -0.12 | 1.57 | 2.20 | 4.87 | 5.13 | 2.78 | 6.48 | 6.06 |
| -3.81 | -5.25 | -1.25 | 1.72 | 4.14 | -1.40 | 5.91 | 6.03 | 2.01 | -0.19 | 3.59 | 0.97 | 0.46 | 1.64 | 4.70 | 3.95 | 2.08 | 5.50 | 5.36 |
| -2.83 | -5.13 | -0.86 | 2.08 | 5.60 | -1.86 | 6.10 | 6.10 | 0.85 | -0.07 | 4.57 | 1.56 | -0.42 | 1.12 | 4.87 | 4.15 | 1.35 | 4.95 | 5.16 |
| -2.07 | -6.04 | -0.61 | 2.54 | 7.31 | -2.56 | 6.16 | 6.39 | -0.35 | -0.18 | 5.66 | 0.75 | -1.16 | 0.40 | 5.27 | 5.32 | 0.46 | 4.84 | 5.26 |
| -2.58 | -7.47 | -0.70 | 3.07 | 8.60 | -3.03 | 5.80 | 6.60 | -1.42 | -0.59 | 6.42 | -1.24 | -1.73 | -0.66 | 5.52 | 6.41 | -0.28 | 4.62 | 5.15 |
| -4.08 | -8.53 | -0.98 | 3.47 | 9.20 | -2.80 | 5.10 | 6.55 | -2.11 | -1.02 | 6.68 | -3.14 | -1.90 | -1.67 | 5.50 | 6.78 | -0.55 | 4.04 | 4.89 |
| -5.68 | -8.68 | -1.27 | 3.56 | 9.26 | -1.90 | 4.53 | 6.32 | -2.15 | -1.10 | 6.64 | -3.95 | -1.57 | -2.24 | 5.51 | 6.69 | -0.27 | 3.11 | 4.87 |
| -6.32 | -8.11 | -1.58 | 3.24 | 8.91 | -1.01 | 4.16 | 5.82 | -1.56 | -0.82 | 6.52 | -3.73 | -1.02 | -2.24 | 5.63 | 6.35 | 0.26 | 2.50 | 5.11 |
| -5.68 | -7.50 | -1.85 | 2.49 | 8.12 | -0.69 | 3.86 | 4.92 | -0.61 | -0.53 | 6.41 | -2.83 | -0.49 | -1.85 | 5.69 | 5.77 | 0.71 | 3.07 | 5.43 |
| -4.32 | -7.09 | -1.92 | 1.72 | 7.31 | -0.75 | 3.64 | 3.87 | 0.27 | -0.50 | 6.28 | -1.69 | -0.26 | -1.20 | 5.71 | 5.12 | 0.87 | 4.70 | 5.67 |

Each cell corresponds to a time instant starting from 5 ms to 1000 ms (y-axis) in every scalp electrode site (x-axis) where the EEG signal was recorded. Averaged-voltage values are in microvolts (μV).

**Table 2. Grand-mean voltages from EEG epochs with correct responses in children with average mathematical achievement level (n=20).**

| **Fp2** | **F4** | **C4** | **P4** | **O2** | **F8** | **T4** | **T6** | **Fz** | **Cz** | **Pz** | **Fp1** | **F3** | **C3** | **P3** | **O1** | **F7** | **T3** | **T5** |
| --- | --- | --- | --- | --- | --- | --- | --- | --- | --- | --- | --- | --- | --- | --- | --- | --- | --- | --- |
| 17.75 | 12.80 | 5.99 | 6.67 | 5.62 | 13.76 | 7.50 | 6.60 | 7.91 | 7.24 | 8.84 | 20.98 | 6.08 | 4.35 | 6.43 | 8.25 | 6.89 | 3.75 | 5.13 |
| 17.73 | 12.85 | 6.29 | 7.04 | 4.62 | 14.55 | 8.39 | 6.96 | 8.08 | 8.07 | 9.66 | 21.05 | 6.58 | 5.21 | 6.32 | 7.61 | 7.10 | 3.99 | 4.85 |
| 18.02 | 12.91 | 6.73 | 7.44 | 3.88 | 15.27 | 9.75 | 7.53 | 8.55 | 8.93 | 10.22 | 21.20 | 7.22 | 5.99 | 6.12 | 6.52 | 7.24 | 4.19 | 4.77 |
| 18.64 | 13.27 | 7.34 | 7.67 | 3.56 | 15.82 | 11.29 | 8.14 | 9.13 | 9.67 | 10.45 | 21.46 | 7.81 | 6.43 | 5.83 | 5.39 | 7.51 | 4.63 | 5.02 |
| 19.52 | 13.94 | 7.93 | 7.48 | 3.30 | 16.20 | 12.63 | 8.34 | 9.71 | 10.04 | 10.19 | 21.96 | 8.09 | 6.54 | 5.32 | 4.33 | 8.06 | 5.64 | 5.16 |
| 20.67 | 14.56 | 8.27 | 6.65 | 2.69 | 16.54 | 13.60 | 7.69 | 10.21 | 9.92 | 9.40 | 22.70 | 8.26 | 6.67 | 4.53 | 3.26 | 8.68 | 7.08 | 4.72 |
| 21.95 | 14.93 | 8.36 | 5.40 | 1.56 | 16.92 | 14.25 | 6.29 | 10.50 | 9.41 | 8.12 | 23.55 | 8.56 | 6.80 | 3.44 | 1.99 | 9.19 | 8.10 | 3.64 |
| 22.72 | 15.09 | 8.37 | 4.10 | 0.24 | 17.21 | 14.69 | 4.64 | 10.56 | 8.72 | 6.61 | 24.33 | 9.05 | 6.94 | 2.23 | 0.57 | 9.61 | 8.20 | 2.38 |
| 22.43 | 15.16 | 8.51 | 3.22 | -0.76 | 17.39 | 15.02 | 3.40 | 10.42 | 8.14 | 5.47 | 24.83 | 9.46 | 7.13 | 1.26 | -0.73 | 10.09 | 8.07 | 1.59 |
| 21.16 | 14.90 | 8.87 | 3.09 | -0.95 | 17.56 | 14.98 | 2.98 | 10.22 | 7.88 | 5.16 | 24.66 | 9.39 | 7.57 | 0.88 | -1.66 | 10.57 | 8.43 | 1.41 |
| 19.72 | 14.33 | 9.31 | 3.68 | -0.33 | 17.83 | 14.47 | 3.45 | 10.02 | 7.88 | 5.71 | 23.49 | 8.72 | 8.20 | 1.22 | -1.97 | 10.56 | 8.96 | 1.62 |
| 18.72 | 13.73 | 9.73 | 4.86 | 0.69 | 18.17 | 13.96 | 4.32 | 9.65 | 8.09 | 6.64 | 21.52 | 7.74 | 8.72 | 1.95 | -1.72 | 9.81 | 8.89 | 1.84 |
| 18.32 | 13.74 | 10.42 | 6.39 | 1.95 | 18.59 | 14.13 | 5.40 | 9.20 | 8.60 | 7.77 | 19.95 | 6.96 | 8.99 | 2.88 | -1.06 | 8.84 | 8.11 | 2.06 |
| 18.44 | 14.66 | 11.66 | 8.36 | 3.61 | 19.12 | 15.03 | 6.63 | 9.05 | 9.74 | 9.21 | 19.79 | 6.86 | 9.31 | 4.16 | -0.16 | 8.53 | 7.24 | 2.57 |
| 19.02 | 16.07 | 13.40 | 10.46 | 5.64 | 19.50 | 15.87 | 7.88 | 9.47 | 11.40 | 10.93 | 20.97 | 7.60 | 10.32 | 5.89 | 0.86 | 9.28 | 7.05 | 3.53 |
| 19.83 | 17.17 | 15.03 | 12.20 | 7.60 | 19.51 | 16.21 | 8.87 | 10.18 | 13.01 | 12.41 | 22.38 | 8.71 | 11.88 | 7.69 | 1.88 | 10.57 | 7.47 | 4.43 |
| 20.65 | 17.45 | 16.08 | 13.23 | 8.97 | 19.18 | 16.29 | 9.31 | 10.71 | 13.98 | 13.24 | 23.21 | 9.64 | 13.34 | 8.93 | 2.70 | 11.65 | 7.83 | 4.97 |
| 21.20 | 17.07 | 16.73 | 13.55 | 9.66 | 19.02 | 16.40 | 9.20 | 10.71 | 14.24 | 13.47 | 23.42 | 10.02 | 14.09 | 9.47 | 3.33 | 12.31 | 7.86 | 4.99 |
| 21.02 | 16.41 | 17.23 | 13.53 | 10.08 | 19.10 | 16.32 | 9.23 | 10.33 | 14.13 | 13.57 | 23.12 | 9.75 | 14.20 | 9.53 | 3.94 | 12.60 | 7.72 | 4.70 |
| 19.75 | 15.81 | 17.59 | 13.39 | 10.41 | 19.09 | 15.93 | 9.90 | 9.78 | 13.92 | 13.84 | 22.08 | 9.07 | 14.12 | 9.38 | 4.62 | 12.84 | 7.82 | 4.30 |
| 17.63 | 15.27 | 17.54 | 13.28 | 10.70 | 18.57 | 15.49 | 11.15 | 9.24 | 13.69 | 14.16 | 20.55 | 8.26 | 14.21 | 9.02 | 5.32 | 13.18 | 8.56 | 4.07 |
| 15.66 | 14.84 | 16.92 | 13.25 | 10.98 | 17.65 | 15.35 | 12.31 | 8.85 | 13.42 | 14.43 | 19.30 | 7.79 | 14.32 | 8.54 | 6.07 | 13.42 | 9.82 | 4.39 |
| 14.75 | 14.82 | 16.08 | 13.12 | 11.11 | 16.66 | 15.41 | 12.95 | 8.91 | 13.28 | 14.64 | 19.03 | 8.06 | 14.25 | 8.27 | 6.96 | 13.41 | 10.87 | 5.51 |
| 15.02 | 15.42 | 15.45 | 13.02 | 11.01 | 15.82 | 15.34 | 13.25 | 9.62 | 13.27 | 14.89 | 19.68 | 9.20 | 14.15 | 8.50 | 8.04 | 13.23 | 10.92 | 7.15 |
| 16.21 | 16.22 | 15.25 | 12.99 | 10.79 | 15.31 | 15.10 | 13.64 | 10.70 | 13.34 | 15.12 | 20.74 | 11.04 | 14.16 | 9.05 | 9.03 | 13.20 | 9.95 | 8.64 |
| 17.74 | 16.68 | 15.22 | 13.06 | 10.53 | 15.13 | 14.51 | 14.20 | 11.44 | 13.17 | 14.97 | 21.58 | 12.76 | 14.20 | 9.51 | 9.45 | 13.48 | 8.63 | 9.38 |
| 19.11 | 16.49 | 15.09 | 13.14 | 10.22 | 15.23 | 13.55 | 14.70 | 11.33 | 12.61 | 14.35 | 21.79 | 13.27 | 14.04 | 9.48 | 9.15 | 13.90 | 7.82 | 9.28 |
| 19.90 | 15.75 | 14.79 | 12.98 | 9.66 | 15.33 | 12.66 | 15.00 | 10.35 | 11.68 | 13.46 | 21.49 | 12.18 | 13.44 | 8.84 | 8.32 | 14.23 | 7.83 | 8.61 |
| 19.73 | 14.85 | 14.40 | 12.46 | 8.89 | 15.33 | 12.67 | 15.02 | 8.93 | 10.53 | 12.46 | 20.92 | 10.23 | 12.52 | 7.83 | 7.14 | 14.19 | 8.26 | 7.84 |
| 18.71 | 14.03 | 13.85 | 11.43 | 8.14 | 15.50 | 13.65 | 14.65 | 7.62 | 9.33 | 11.45 | 20.15 | 8.65 | 11.52 | 6.70 | 5.75 | 13.64 | 8.57 | 7.30 |
| 17.45 | 13.55 | 13.15 | 10.12 | 7.51 | 16.03 | 14.83 | 13.91 | 6.79 | 8.33 | 10.34 | 19.24 | 8.05 | 10.57 | 5.68 | 4.35 | 12.74 | 8.46 | 7.01 |
| 16.79 | 13.49 | 12.50 | 9.07 | 7.10 | 16.74 | 15.53 | 13.24 | 6.42 | 7.48 | 9.11 | 18.27 | 8.20 | 9.62 | 4.89 | 3.15 | 11.92 | 8.06 | 6.84 |
| 16.84 | 13.73 | 12.12 | 8.62 | 6.84 | 17.10 | 15.62 | 12.95 | 6.32 | 6.78 | 7.88 | 17.52 | 8.67 | 8.70 | 4.16 | 2.34 | 11.44 | 7.50 | 6.41 |
| 16.91 | 13.98 | 12.03 | 8.77 | 6.38 | 16.92 | 15.42 | 12.93 | 6.26 | 6.09 | 6.88 | 17.17 | 9.12 | 7.79 | 3.36 | 1.65 | 11.42 | 6.62 | 5.40 |
| 16.65 | 14.07 | 12.11 | 9.34 | 5.71 | 16.58 | 14.95 | 12.87 | 6.33 | 5.50 | 6.27 | 17.15 | 9.51 | 7.18 | 2.71 | 1.04 | 11.93 | 5.55 | 4.10 |
| 16.57 | 14.01 | 12.39 | 10.02 | 5.19 | 16.56 | 13.96 | 12.66 | 6.74 | 5.24 | 6.24 | 17.35 | 9.77 | 7.18 | 2.51 | 0.67 | 12.93 | 4.89 | 3.15 |
| 17.28 | 13.93 | 12.78 | 10.58 | 5.26 | 16.83 | 12.74 | 12.28 | 7.47 | 5.50 | 6.75 | 17.80 | 9.86 | 7.79 | 3.02 | 0.54 | 14.13 | 4.78 | 2.87 |
| 18.87 | 14.07 | 13.28 | 10.90 | 6.08 | 17.23 | 12.14 | 11.92 | 8.40 | 6.24 | 7.55 | 18.49 | 9.89 | 8.65 | 4.16 | 0.80 | 15.08 | 5.10 | 3.24 |
| 20.51 | 14.44 | 13.98 | 11.08 | 7.24 | 17.60 | 12.81 | 11.75 | 9.42 | 7.41 | 8.33 | 19.49 | 10.12 | 9.48 | 5.59 | 1.58 | 15.43 | 5.68 | 3.98 |
| 21.37 | 14.80 | 14.86 | 11.23 | 8.21 | 18.05 | 14.39 | 11.77 | 10.26 | 8.75 | 8.95 | 20.47 | 10.53 | 10.05 | 6.92 | 2.62 | 15.20 | 6.33 | 4.80 |
| 21.31 | 15.05 | 15.70 | 11.37 | 8.67 | 18.55 | 15.71 | 11.71 | 10.71 | 9.98 | 9.30 | 20.62 | 11.04 | 10.38 | 7.78 | 3.67 | 14.57 | 6.93 | 5.38 |
| 20.82 | 15.19 | 16.09 | 11.27 | 8.38 | 18.93 | 15.82 | 11.26 | 10.69 | 10.74 | 9.28 | 19.52 | 11.41 | 10.41 | 8.06 | 4.20 | 13.78 | 7.22 | 5.50 |
| 20.27 | 15.16 | 15.88 | 10.77 | 7.57 | 18.94 | 14.83 | 10.48 | 10.52 | 11.08 | 8.77 | 17.59 | 11.63 | 10.18 | 7.87 | 4.22 | 13.00 | 7.23 | 5.01 |
| 19.40 | 14.81 | 15.28 | 10.11 | 6.78 | 18.59 | 13.65 | 9.77 | 10.57 | 11.30 | 7.97 | 15.94 | 11.55 | 9.90 | 7.74 | 4.14 | 12.28 | 7.19 | 4.06 |
| 18.29 | 14.01 | 14.60 | 9.55 | 6.48 | 18.09 | 12.84 | 9.25 | 10.83 | 11.61 | 7.25 | 15.36 | 11.24 | 9.89 | 8.05 | 4.14 | 11.59 | 6.98 | 3.01 |
| 17.27 | 12.60 | 14.01 | 9.35 | 6.63 | 17.63 | 12.31 | 8.83 | 10.81 | 11.92 | 6.78 | 15.62 | 10.72 | 10.10 | 8.90 | 4.33 | 10.90 | 6.56 | 2.45 |
| 16.65 | 10.65 | 13.40 | 9.38 | 6.98 | 17.11 | 11.51 | 8.42 | 10.21 | 11.98 | 6.65 | 15.79 | 10.06 | 10.35 | 10.30 | 4.71 | 10.37 | 6.09 | 2.87 |
| 16.20 | 8.43 | 12.66 | 9.50 | 7.21 | 16.29 | 10.17 | 8.06 | 8.99 | 11.61 | 6.82 | 15.21 | 9.24 | 10.30 | 12.00 | 5.24 | 10.18 | 5.85 | 4.28 |
| 15.82 | 6.47 | 11.81 | 9.48 | 7.31 | 15.18 | 9.13 | 7.79 | 7.50 | 11.04 | 7.17 | 14.25 | 8.11 | 9.87 | 13.72 | 5.80 | 10.22 | 5.83 | 6.09 |
| 15.55 | 5.30 | 10.96 | 9.44 | 7.27 | 14.23 | 9.26 | 7.68 | 6.19 | 10.52 | 7.74 | 13.36 | 6.79 | 9.26 | 15.11 | 6.02 | 10.05 | 6.12 | 7.57 |
| 15.41 | 5.22 | 10.51 | 9.74 | 7.46 | 13.68 | 10.57 | 7.93 | 5.53 | 10.36 | 8.58 | 12.53 | 5.93 | 8.83 | 16.09 | 5.81 | 9.47 | 6.97 | 8.35 |
| 15.13 | 6.28 | 10.60 | 10.37 | 7.88 | 13.57 | 12.38 | 8.49 | 5.79 | 10.50 | 9.48 | 11.79 | 5.97 | 8.74 | 16.83 | 5.41 | 8.78 | 8.07 | 8.53 |
| 14.73 | 7.98 | 11.00 | 10.97 | 8.32 | 13.71 | 13.82 | 8.91 | 6.75 | 10.73 | 10.29 | 11.22 | 6.82 | 9.09 | 17.52 | 5.25 | 8.70 | 8.80 | 8.58 |
| 14.77 | 9.63 | 11.17 | 11.14 | 8.63 | 14.04 | 14.49 | 8.86 | 8.22 | 10.86 | 10.80 | 11.27 | 8.14 | 9.76 | 18.32 | 5.59 | 9.44 | 8.77 | 9.03 |
| 15.31 | 10.50 | 10.76 | 10.66 | 8.70 | 14.40 | 14.29 | 8.37 | 9.65 | 10.77 | 10.91 | 12.15 | 9.33 | 10.42 | 19.17 | 6.12 | 10.51 | 8.59 | 10.02 |
| 15.71 | 10.43 | 9.93 | 9.73 | 8.67 | 14.68 | 13.20 | 7.95 | 10.72 | 10.62 | 10.75 | 13.51 | 10.17 | 10.84 | 19.97 | 6.47 | 11.45 | 8.91 | 11.23 |
| 15.43 | 9.87 | 9.13 | 8.83 | 8.84 | 14.66 | 11.54 | 7.91 | 11.39 | 10.42 | 10.67 | 14.82 | 10.87 | 10.94 | 20.83 | 6.67 | 12.03 | 9.55 | 12.29 |
| 14.90 | 9.40 | 8.75 | 8.44 | 9.52 | 14.31 | 10.39 | 8.12 | 11.70 | 10.38 | 10.83 | 15.70 | 11.51 | 11.05 | 21.47 | 7.05 | 12.24 | 9.78 | 12.90 |
| 14.94 | 9.17 | 8.90 | 8.78 | 10.66 | 13.83 | 10.19 | 8.40 | 11.75 | 10.55 | 11.28 | 16.21 | 12.01 | 11.37 | 22.34 | 7.89 | 12.35 | 9.37 | 13.07 |
| 15.54 | 8.87 | 9.33 | 9.70 | 11.84 | 13.47 | 10.49 | 8.42 | 11.44 | 10.88 | 11.77 | 16.43 | 12.03 | 11.85 | 23.30 | 8.92 | 12.61 | 8.68 | 12.80 |
| 16.04 | 8.21 | 9.79 | 10.71 | 12.27 | 13.30 | 10.62 | 7.95 | 10.69 | 11.19 | 12.00 | 16.21 | 11.45 | 12.21 | 23.93 | 9.61 | 13.01 | 8.31 | 12.12 |
| 16.17 | 7.38 | 10.24 | 11.42 | 11.83 | 13.23 | 10.45 | 7.28 | 9.76 | 11.43 | 11.99 | 15.29 | 10.56 | 12.28 | 24.14 | 9.81 | 13.33 | 8.51 | 11.14 |
| 16.24 | 6.73 | 10.75 | 11.65 | 11.04 | 13.29 | 10.26 | 6.88 | 9.01 | 11.66 | 12.00 | 13.84 | 9.67 | 12.18 | 24.08 | 9.95 | 13.19 | 9.05 | 10.23 |
| 16.59 | 6.59 | 11.37 | 11.55 | 10.49 | 13.72 | 10.40 | 6.97 | 8.74 | 11.89 | 12.27 | 12.60 | 9.11 | 12.07 | 23.88 | 10.39 | 12.67 | 9.67 | 9.61 |
| 17.03 | 7.21 | 12.08 | 11.20 | 10.50 | 14.48 | 10.71 | 7.25 | 8.98 | 12.05 | 12.73 | 12.41 | 9.04 | 12.01 | 23.49 | 11.09 | 12.07 | 10.03 | 9.25 |
| 17.07 | 8.48 | 12.68 | 10.67 | 10.73 | 15.17 | 10.91 | 7.38 | 9.47 | 12.13 | 13.10 | 13.03 | 9.51 | 11.88 | 22.88 | 11.61 | 11.54 | 10.04 | 8.98 |
| 16.53 | 9.98 | 12.97 | 9.93 | 10.85 | 15.20 | 10.82 | 7.13 | 9.85 | 12.02 | 13.05 | 13.46 | 10.24 | 11.72 | 22.13 | 11.54 | 11.03 | 9.87 | 8.70 |
| 15.55 | 11.08 | 12.93 | 8.94 | 10.72 | 14.92 | 10.19 | 6.63 | 9.95 | 11.66 | 12.54 | 12.73 | 10.88 | 11.42 | 21.26 | 10.79 | 10.57 | 9.61 | 8.22 |
| 14.60 | 11.40 | 12.64 | 7.82 | 10.29 | 15.01 | 9.20 | 6.17 | 9.67 | 10.94 | 11.52 | 10.82 | 11.07 | 10.94 | 20.23 | 9.50 | 10.19 | 8.97 | 7.58 |
| 14.17 | 11.08 | 12.25 | 6.75 | 9.52 | 15.67 | 8.36 | 6.03 | 9.19 | 9.86 | 10.00 | 8.84 | 10.53 | 10.12 | 19.00 | 7.91 | 9.92 | 7.38 | 6.83 |
| 14.62 | 10.59 | 11.64 | 6.01 | 8.47 | 16.73 | 7.99 | 6.19 | 8.52 | 8.57 | 8.38 | 7.75 | 9.31 | 8.95 | 17.68 | 6.52 | 9.78 | 5.08 | 6.19 |
| 15.75 | 10.27 | 10.77 | 5.82 | 7.52 | 17.80 | 8.04 | 6.52 | 7.69 | 7.35 | 7.09 | 8.00 | 7.77 | 7.53 | 16.63 | 5.67 | 9.65 | 3.22 | 5.87 |
| 16.95 | 10.19 | 9.62 | 6.13 | 7.04 | 18.63 | 8.45 | 6.90 | 6.83 | 6.47 | 6.33 | 9.30 | 6.65 | 6.25 | 16.08 | 5.36 | 9.52 | 2.89 | 5.84 |
| 17.38 | 10.16 | 8.47 | 6.72 | 7.02 | 18.99 | 9.08 | 7.20 | 6.16 | 5.97 | 6.01 | 10.94 | 6.31 | 5.32 | 16.08 | 5.29 | 9.46 | 3.90 | 5.88 |
| 16.99 | 10.07 | 7.47 | 7.30 | 7.22 | 18.61 | 9.71 | 7.66 | 5.79 | 5.83 | 5.95 | 12.20 | 6.61 | 4.90 | 16.18 | 5.15 | 9.60 | 4.93 | 5.87 |
| 16.37 | 9.76 | 6.77 | 7.85 | 7.50 | 17.64 | 9.94 | 8.35 | 5.73 | 6.01 | 6.09 | 12.52 | 7.32 | 4.95 | 16.32 | 4.94 | 9.97 | 5.33 | 6.14 |
| 15.93 | 9.25 | 6.39 | 8.47 | 7.81 | 16.47 | 9.44 | 9.11 | 5.86 | 6.52 | 6.51 | 12.10 | 8.34 | 5.40 | 16.46 | 4.72 | 10.60 | 5.68 | 6.78 |
| 15.76 | 8.78 | 6.47 | 9.05 | 8.11 | 15.33 | 8.51 | 9.75 | 6.14 | 7.25 | 6.98 | 11.72 | 9.48 | 6.07 | 16.71 | 4.73 | 11.39 | 6.61 | 7.61 |
| 15.71 | 8.65 | 6.83 | 9.46 | 8.05 | 14.28 | 7.92 | 10.02 | 6.40 | 8.10 | 7.35 | 11.90 | 10.43 | 6.82 | 17.08 | 5.10 | 12.01 | 7.98 | 8.43 |
| 15.77 | 8.88 | 7.14 | 9.36 | 7.25 | 13.45 | 7.92 | 9.72 | 6.55 | 8.82 | 7.62 | 12.69 | 10.96 | 7.46 | 17.53 | 5.66 | 12.24 | 9.24 | 8.94 |
| 15.85 | 9.19 | 7.25 | 8.71 | 5.93 | 12.97 | 8.18 | 8.86 | 6.52 | 9.36 | 7.90 | 13.47 | 11.09 | 8.04 | 18.04 | 6.36 | 12.30 | 10.22 | 9.17 |
| 15.51 | 9.19 | 7.07 | 7.53 | 4.42 | 12.92 | 8.06 | 7.70 | 6.28 | 9.64 | 8.15 | 13.49 | 10.84 | 8.51 | 18.50 | 7.12 | 12.44 | 10.91 | 9.33 |
| 14.52 | 8.92 | 6.91 | 6.12 | 3.15 | 13.03 | 7.35 | 6.69 | 5.95 | 9.70 | 8.28 | 12.92 | 10.31 | 8.62 | 18.87 | 7.90 | 12.64 | 11.41 | 9.65 |
| 13.44 | 8.81 | 7.15 | 5.01 | 2.37 | 13.13 | 6.67 | 6.09 | 5.79 | 9.62 | 8.23 | 12.43 | 9.61 | 8.41 | 19.23 | 8.69 | 12.55 | 11.88 | 10.35 |
| 12.76 | 9.15 | 7.92 | 4.56 | 2.19 | 13.13 | 6.61 | 5.94 | 5.86 | 9.50 | 8.07 | 12.17 | 8.96 | 8.03 | 19.58 | 9.30 | 11.92 | 12.13 | 11.41 |
| 13.01 | 9.82 | 8.92 | 4.93 | 2.88 | 13.21 | 7.26 | 6.22 | 5.91 | 9.35 | 7.94 | 11.82 | 8.50 | 7.66 | 19.78 | 9.77 | 10.88 | 11.89 | 12.53 |
| 13.91 | 10.33 | 9.67 | 5.80 | 4.25 | 13.61 | 8.16 | 7.04 | 5.63 | 9.12 | 7.96 | 11.01 | 8.21 | 7.28 | 19.65 | 10.10 | 10.19 | 11.15 | 13.20 |
| 14.69 | 10.43 | 9.95 | 6.81 | 5.78 | 14.34 | 8.63 | 8.24 | 5.11 | 8.92 | 7.99 | 10.05 | 7.94 | 6.88 | 19.14 | 10.27 | 10.27 | 10.19 | 13.03 |
| 14.79 | 10.32 | 9.84 | 7.74 | 6.98 | 15.13 | 8.58 | 9.49 | 4.65 | 8.93 | 7.89 | 9.71 | 7.63 | 6.68 | 18.57 | 10.32 | 10.88 | 9.35 | 12.21 |
| 14.39 | 10.09 | 9.47 | 8.53 | 7.62 | 15.43 | 8.46 | 10.15 | 4.60 | 9.25 | 7.73 | 10.32 | 7.38 | 6.77 | 18.28 | 10.30 | 11.43 | 9.03 | 11.10 |
| 13.79 | 9.65 | 8.84 | 9.03 | 7.67 | 14.93 | 8.68 | 9.90 | 4.86 | 9.60 | 7.50 | 11.39 | 7.37 | 6.89 | 18.06 | 10.02 | 11.55 | 9.10 | 9.89 |
| 13.06 | 8.82 | 7.87 | 9.10 | 7.28 | 13.86 | 9.35 | 8.92 | 5.03 | 9.53 | 7.17 | 12.21 | 7.48 | 6.51 | 17.50 | 9.14 | 11.31 | 9.18 | 8.55 |
| 12.26 | 7.83 | 6.95 | 8.90 | 7.01 | 12.80 | 10.11 | 7.96 | 4.95 | 8.99 | 6.84 | 12.68 | 7.56 | 5.61 | 16.54 | 7.98 | 11.08 | 9.05 | 7.13 |
| 11.92 | 7.24 | 6.37 | 8.74 | 7.29 | 12.19 | 10.29 | 7.66 | 4.60 | 8.14 | 6.66 | 12.94 | 7.51 | 4.60 | 15.49 | 7.26 | 10.78 | 8.83 | 5.80 |
| 12.15 | 7.27 | 6.30 | 9.00 | 8.13 | 12.24 | 9.59 | 8.14 | 4.31 | 7.24 | 6.85 | 13.06 | 7.53 | 4.10 | 14.90 | 7.31 | 10.23 | 8.81 | 4.71 |
| 12.71 | 7.65 | 6.82 | 9.73 | 9.04 | 12.71 | 8.61 | 8.98 | 4.27 | 6.67 | 7.52 | 12.93 | 7.87 | 4.37 | 14.83 | 7.95 | 9.65 | 9.29 | 3.81 |
| 13.11 | 8.14 | 7.71 | 10.60 | 9.44 | 13.27 | 8.39 | 9.59 | 4.55 | 6.65 | 8.45 | 12.62 | 8.54 | 5.33 | 14.77 | 8.37 | 9.26 | 9.94 | 3.20 |
| 13.47 | 8.79 | 8.75 | 11.24 | 9.15 | 13.64 | 9.07 | 9.80 | 5.16 | 7.09 | 9.15 | 12.52 | 9.31 | 6.61 | 14.29 | 8.05 | 9.24 | 10.41 | 2.87 |
| 14.11 | 9.68 | 9.52 | 11.31 | 8.48 | 13.85 | 9.50 | 9.44 | 5.94 | 7.77 | 9.30 | 12.90 | 9.93 | 7.55 | 13.20 | 7.08 | 9.49 | 10.58 | 2.71 |
| 14.88 | 10.67 | 9.73 | 10.72 | 7.68 | 14.16 | 9.14 | 8.54 | 6.64 | 8.25 | 8.75 | 13.38 | 10.43 | 8.04 | 11.73 | 5.91 | 9.67 | 10.58 | 2.57 |
| 15.04 | 11.23 | 9.45 | 9.51 | 6.81 | 14.60 | 8.88 | 7.32 | 7.13 | 8.43 | 7.79 | 13.19 | 10.77 | 8.03 | 10.30 | 4.95 | 9.37 | 10.82 | 2.44 |
| 14.53 | 10.88 | 8.93 | 7.93 | 5.89 | 14.94 | 9.53 | 6.12 | 7.31 | 8.24 | 6.57 | 12.08 | 10.68 | 7.52 | 9.10 | 4.20 | 8.49 | 11.54 | 2.41 |
| 13.86 | 9.82 | 8.34 | 6.30 | 5.05 | 15.00 | 10.65 | 5.20 | 7.18 | 7.79 | 5.29 | 10.83 | 10.06 | 6.66 | 7.99 | 3.36 | 7.35 | 12.30 | 2.58 |
| 13.68 | 8.68 | 7.80 | 5.04 | 4.60 | 14.88 | 11.09 | 4.73 | 6.85 | 7.26 | 4.10 | 10.75 | 9.09 | 5.66 | 6.80 | 2.46 | 6.42 | 12.37 | 2.84 |
| 14.11 | 8.11 | 7.19 | 4.16 | 4.49 | 14.60 | 10.66 | 4.73 | 6.63 | 6.87 | 3.12 | 12.15 | 8.21 | 4.82 | 5.69 | 1.92 | 6.18 | 11.57 | 3.09 |
| 14.77 | 8.45 | 6.57 | 3.64 | 4.62 | 14.15 | 10.16 | 5.18 | 6.66 | 6.77 | 2.43 | 13.94 | 7.80 | 4.37 | 5.01 | 1.97 | 6.60 | 10.35 | 3.30 |
| 14.99 | 9.30 | 6.10 | 3.44 | 4.92 | 13.52 | 9.88 | 5.90 | 6.90 | 6.87 | 2.00 | 14.60 | 7.70 | 4.29 | 4.93 | 2.51 | 7.01 | 9.58 | 3.63 |
| 14.30 | 10.04 | 5.77 | 3.44 | 5.41 | 12.74 | 9.32 | 6.47 | 7.04 | 6.86 | 1.90 | 13.58 | 7.63 | 4.34 | 5.28 | 3.22 | 6.77 | 9.54 | 4.23 |
| 12.91 | 10.26 | 5.58 | 3.66 | 6.26 | 12.18 | 8.54 | 6.85 | 6.75 | 6.33 | 2.02 | 11.49 | 7.36 | 4.11 | 5.82 | 4.07 | 5.91 | 9.94 | 5.03 |
| 11.62 | 10.14 | 5.54 | 4.23 | 7.33 | 11.92 | 8.24 | 7.25 | 6.14 | 5.52 | 2.32 | 9.52 | 6.91 | 3.64 | 6.41 | 5.17 | 5.10 | 10.79 | 6.05 |
| 11.24 | 9.95 | 5.79 | 5.43 | 8.56 | 11.80 | 9.02 | 8.08 | 5.53 | 4.89 | 3.02 | 8.44 | 6.56 | 3.35 | 7.31 | 6.77 | 4.74 | 12.09 | 7.25 |
| 11.89 | 9.88 | 6.26 | 7.41 | 9.97 | 11.66 | 10.27 | 9.53 | 5.11 | 4.76 | 4.37 | 8.29 | 6.33 | 3.63 | 8.68 | 8.80 | 4.58 | 13.47 | 8.61 |
| 12.81 | 9.67 | 6.64 | 9.88 | 11.48 | 11.56 | 10.79 | 11.35 | 4.87 | 4.91 | 6.22 | 8.63 | 6.21 | 4.36 | 10.40 | 11.08 | 4.26 | 14.38 | 9.88 |
| 12.91 | 9.10 | 6.64 | 12.16 | 12.85 | 11.60 | 10.12 | 12.98 | 4.61 | 4.91 | 8.17 | 9.11 | 6.16 | 4.95 | 11.93 | 13.21 | 4.03 | 14.68 | 10.64 |
| 12.05 | 8.14 | 6.21 | 13.62 | 13.72 | 11.93 | 8.95 | 13.88 | 4.31 | 4.54 | 9.63 | 9.45 | 5.92 | 5.06 | 12.95 | 14.64 | 4.21 | 14.54 | 10.69 |
| 10.65 | 7.00 | 5.81 | 14.19 | 13.83 | 12.46 | 8.21 | 14.07 | 4.05 | 3.95 | 10.31 | 9.35 | 5.53 | 4.75 | 13.46 | 15.06 | 4.50 | 14.04 | 10.37 |
| 9.55 | 5.98 | 5.57 | 13.84 | 13.05 | 12.80 | 7.95 | 13.78 | 3.72 | 3.23 | 10.05 | 8.61 | 4.84 | 4.25 | 13.41 | 14.26 | 4.23 | 12.75 | 9.95 |
| 8.96 | 5.29 | 5.56 | 12.69 | 11.49 | 12.64 | 7.57 | 13.20 | 3.36 | 2.51 | 8.90 | 7.47 | 3.92 | 3.66 | 12.73 | 12.52 | 3.44 | 10.96 | 9.39 |
| 8.59 | 4.92 | 5.49 | 10.99 | 9.69 | 11.71 | 6.67 | 12.42 | 2.97 | 1.89 | 7.16 | 6.21 | 3.04 | 3.03 | 11.52 | 10.44 | 2.42 | 9.62 | 8.52 |
| 7.94 | 4.66 | 5.09 | 9.04 | 8.16 | 10.09 | 5.53 | 11.40 | 2.39 | 1.30 | 5.02 | 4.91 | 2.31 | 2.31 | 9.95 | 8.52 | 1.37 | 9.07 | 7.33 |
| 6.79 | 4.20 | 4.27 | 7.26 | 7.14 | 8.25 | 4.67 | 10.27 | 1.50 | 0.70 | 3.01 | 3.62 | 1.67 | 1.77 | 8.40 | 7.26 | 0.50 | 8.87 | 6.43 |
| 5.33 | 3.59 | 3.23 | 5.86 | 6.45 | 6.82 | 4.32 | 9.22 | 0.42 | 0.12 | 1.38 | 2.52 | 0.94 | 1.59 | 7.10 | 6.83 | -0.08 | 8.73 | 6.32 |
| 3.99 | 3.13 | 2.30 | 4.91 | 5.93 | 5.90 | 4.47 | 8.30 | -0.44 | -0.43 | 0.42 | 2.03 | 0.23 | 1.80 | 6.16 | 7.07 | -0.22 | 8.77 | 6.84 |
| 3.22 | 3.13 | 1.78 | 4.26 | 5.72 | 5.53 | 4.83 | 7.45 | -0.72 | -0.90 | 0.09 | 2.28 | -0.07 | 2.06 | 5.62 | 7.73 | 0.06 | 9.22 | 7.53 |
| 3.23 | 3.44 | 1.61 | 3.65 | 5.81 | 5.62 | 4.88 | 6.51 | -0.29 | -1.16 | 0.12 | 3.11 | 0.15 | 2.19 | 5.30 | 8.45 | 0.67 | 10.42 | 8.15 |
| 3.79 | 3.70 | 1.50 | 2.84 | 5.69 | 6.09 | 4.15 | 5.45 | 0.48 | -1.24 | 0.02 | 4.16 | 0.62 | 2.17 | 4.89 | 8.84 | 1.39 | 11.84 | 8.43 |
| 4.47 | 3.56 | 1.36 | 1.83 | 5.22 | 6.62 | 2.78 | 4.45 | 1.24 | -1.09 | -0.47 | 4.71 | 1.01 | 2.20 | 4.05 | 8.68 | 1.93 | 12.69 | 8.24 |
| 5.07 | 2.98 | 1.24 | 1.02 | 4.54 | 6.96 | 1.61 | 3.72 | 1.67 | -0.77 | -1.31 | 4.61 | 1.24 | 2.34 | 2.89 | 8.14 | 2.04 | 12.32 | 7.59 |
| 5.67 | 2.40 | 1.42 | 0.72 | 3.88 | 7.04 | 1.43 | 3.31 | 1.73 | -0.43 | -2.33 | 4.47 | 1.69 | 2.63 | 1.67 | 7.62 | 1.99 | 11.28 | 6.79 |
| 6.27 | 2.15 | 1.89 | 0.74 | 3.20 | 7.04 | 2.11 | 2.99 | 1.65 | -0.22 | -3.41 | 4.96 | 2.52 | 2.97 | 0.56 | 7.23 | 2.21 | 10.82 | 6.09 |
| 6.41 | 2.43 | 2.46 | 0.74 | 2.18 | 6.87 | 3.03 | 2.54 | 1.70 | -0.06 | -4.48 | 5.84 | 3.27 | 3.29 | -0.42 | 6.60 | 2.61 | 11.55 | 5.55 |
| 5.79 | 3.11 | 2.97 | 0.39 | 0.89 | 6.54 | 3.63 | 1.89 | 1.84 | 0.16 | -5.46 | 6.32 | 3.22 | 3.53 | -1.45 | 5.23 | 2.77 | 12.41 | 4.91 |
| 4.59 | 3.88 | 3.25 | -0.32 | -0.19 | 6.28 | 3.99 | 0.98 | 1.91 | 0.44 | -6.03 | 6.06 | 2.22 | 3.66 | -2.44 | 3.50 | 2.46 | 12.20 | 4.10 |
| 3.77 | 4.12 | 3.25 | -0.90 | -0.52 | 6.36 | 4.67 | -0.07 | 1.57 | 0.73 | -6.08 | 5.66 | 0.83 | 3.97 | -3.17 | 2.19 | 1.83 | 10.85 | 3.46 |
| 3.96 | 3.62 | 3.22 | -0.95 | 0.09 | 6.93 | 5.63 | -0.81 | 0.88 | 1.01 | -5.48 | 5.84 | -0.04 | 4.62 | -3.49 | 1.88 | 1.39 | 9.44 | 3.14 |
| 5.11 | 2.71 | 3.48 | -0.40 | 1.29 | 7.83 | 6.73 | -1.04 | 0.44 | 1.40 | -4.54 | 6.90 | 0.08 | 5.52 | -3.44 | 2.29 | 1.32 | 9.09 | 3.00 |
| 6.40 | 2.20 | 4.19 | 0.33 | 2.46 | 8.90 | 7.95 | -0.87 | 0.83 | 1.92 | -3.68 | 8.12 | 1.15 | 6.21 | -3.46 | 2.53 | 1.65 | 10.04 | 2.69 |
| 7.33 | 2.59 | 5.11 | 0.73 | 3.09 | 10.16 | 9.17 | -0.69 | 2.07 | 2.55 | -3.25 | 8.73 | 2.90 | 6.43 | -3.88 | 1.83 | 2.35 | 11.25 | 2.02 |
| 8.05 | 3.76 | 5.83 | 0.63 | 3.19 | 11.56 | 9.90 | -0.76 | 3.71 | 3.03 | -3.28 | 8.79 | 4.91 | 6.30 | -4.66 | 0.32 | 3.41 | 11.61 | 1.01 |
| 8.96 | 5.34 | 6.16 | 0.35 | 3.26 | 12.81 | 9.64 | -0.89 | 5.31 | 3.52 | -3.47 | 8.88 | 6.81 | 6.25 | -5.39 | -1.26 | 4.61 | 11.08 | 0.13 |
| 10.02 | 6.89 | 6.30 | 0.43 | 3.74 | 13.37 | 8.53 | -0.68 | 6.80 | 4.29 | -3.28 | 9.39 | 8.38 | 6.82 | -5.53 | -1.98 | 5.81 | 10.62 | -0.13 |
| 10.28 | 7.99 | 6.19 | 0.81 | 4.55 | 12.86 | 7.11 | -0.30 | 8.09 | 5.28 | -2.63 | 9.72 | 9.25 | 7.97 | -5.10 | -1.75 | 6.58 | 11.10 | 0.32 |
| 9.51 | 8.44 | 5.63 | 0.93 | 5.12 | 11.41 | 5.71 | -0.30 | 8.92 | 6.10 | -1.75 | 9.21 | 9.22 | 9.16 | -4.69 | -1.25 | 6.72 | 12.24 | 1.14 |
| 8.76 | 8.28 | 4.55 | 0.43 | 5.21 | 9.74 | 4.22 | -0.83 | 9.20 | 6.48 | -0.99 | 8.56 | 8.47 | 9.93 | -4.76 | -1.01 | 6.54 | 13.18 | 1.91 |
| 9.12 | 7.76 | 3.29 | -0.61 | 4.81 | 8.56 | 2.62 | -1.73 | 9.19 | 6.39 | -0.51 | 8.90 | 7.67 | 10.21 | -5.20 | -1.11 | 6.83 | 13.53 | 2.47 |
| 10.58 | 7.31 | 2.37 | -1.70 | 4.15 | 7.98 | 1.45 | -2.50 | 9.16 | 6.17 | -0.35 | 10.42 | 7.31 | 10.34 | -5.69 | -1.17 | 8.09 | 13.62 | 3.10 |
| 11.98 | 7.14 | 1.83 | -2.48 | 3.55 | 7.80 | 1.35 | -2.91 | 9.16 | 6.00 | -0.40 | 11.87 | 7.36 | 10.47 | -6.00 | -0.94 | 10.10 | 13.81 | 4.00 |
| 12.36 | 7.15 | 1.62 | -2.97 | 3.25 | 8.05 | 2.34 | -2.99 | 9.25 | 5.89 | -0.70 | 12.24 | 7.66 | 10.53 | -6.22 | -0.43 | 12.08 | 14.35 | 5.19 |
| 11.68 | 7.02 | 1.68 | -3.33 | 3.13 | 8.70 | 3.89 | -2.71 | 9.16 | 5.56 | -1.21 | 11.60 | 8.11 | 10.29 | -6.41 | 0.24 | 13.31 | 15.06 | 6.32 |
| 10.41 | 6.55 | 2.09 | -3.51 | 3.12 | 9.64 | 5.36 | -1.99 | 8.69 | 4.92 | -1.84 | 10.36 | 8.63 | 9.80 | -6.60 | 0.95 | 13.76 | 15.71 | 7.13 |
| 8.94 | 5.91 | 2.80 | -3.20 | 3.29 | 10.48 | 6.42 | -0.91 | 7.89 | 4.07 | -2.41 | 8.93 | 9.15 | 9.22 | -6.68 | 1.61 | 13.77 | 16.47 | 7.57 |
| 7.62 | 5.57 | 3.64 | -2.38 | 3.82 | 11.01 | 6.94 | 0.17 | 7.16 | 3.28 | -2.66 | 7.84 | 9.59 | 8.80 | -6.68 | 2.23 | 13.74 | 17.44 | 7.80 |
| 7.02 | 5.66 | 4.45 | -1.30 | 4.66 | 11.21 | 7.18 | 1.05 | 6.99 | 2.81 | -2.51 | 7.96 | 10.10 | 8.61 | -6.48 | 2.90 | 13.86 | 18.37 | 7.93 |
| 7.54 | 6.06 | 5.18 | -0.37 | 5.58 | 11.27 | 7.46 | 1.76 | 7.44 | 2.64 | -2.19 | 9.50 | 10.43 | 8.60 | -6.10 | 3.51 | 13.85 | 18.80 | 7.89 |
| 9.24 | 6.38 | 5.73 | 0.10 | 6.34 | 11.40 | 8.00 | 2.43 | 8.20 | 2.58 | -2.06 | 11.57 | 10.48 | 8.72 | -5.66 | 3.79 | 13.51 | 18.35 | 7.71 |
| 11.47 | 6.41 | 6.16 | -0.01 | 6.82 | 11.93 | 8.92 | 3.13 | 8.83 | 2.40 | -2.49 | 12.80 | 10.40 | 8.81 | -5.37 | 3.68 | 12.94 | 17.26 | 7.57 |
| 12.89 | 6.19 | 6.42 | -0.60 | 6.57 | 12.78 | 9.98 | 3.52 | 9.09 | 2.01 | -3.54 | 12.49 | 10.41 | 8.69 | -5.55 | 3.09 | 12.42 | 16.55 | 7.73 |
| 12.34 | 5.88 | 6.42 | -1.55 | 5.23 | 13.49 | 10.71 | 3.08 | 8.75 | 1.40 | -4.95 | 11.15 | 10.20 | 8.20 | -6.27 | 1.89 | 12.15 | 16.55 | 8.08 |
| 9.89 | 5.59 | 6.13 | -2.44 | 3.26 | 13.64 | 10.61 | 2.02 | 8.03 | 0.75 | -6.30 | 9.65 | 9.55 | 7.53 | -7.18 | 0.41 | 12.07 | 16.80 | 8.29 |
| 7.00 | 5.35 | 5.62 | -3.07 | 1.47 | 13.33 | 9.65 | 1.12 | 7.21 | 0.33 | -7.31 | 8.69 | 8.68 | 6.92 | -7.86 | -0.99 | 12.08 | 16.73 | 8.19 |
| 5.28 | 5.05 | 4.93 | -3.52 | 0.31 | 12.77 | 8.56 | 0.95 | 6.48 | 0.03 | -8.04 | 8.35 | 8.08 | 6.40 | -8.29 | -2.16 | 12.07 | 16.04 | 7.72 |
| 5.40 | 4.58 | 3.96 | -4.11 | -0.59 | 12.15 | 8.30 | 1.32 | 5.82 | -0.40 | -8.75 | 8.60 | 7.82 | 5.71 | -8.80 | -3.33 | 11.90 | 14.98 | 6.84 |
| 6.93 | 4.02 | 2.92 | -4.83 | -1.74 | 11.53 | 9.26 | 1.54 | 5.22 | -1.04 | -9.45 | 9.15 | 7.66 | 4.79 | -9.54 | -4.64 | 11.68 | 14.06 | 5.47 |
| 8.63 | 3.55 | 2.16 | -5.48 | -3.31 | 10.83 | 10.54 | 1.22 | 4.85 | -1.59 | -9.99 | 9.88 | 7.38 | 3.77 | -10.29 | -5.96 | 11.65 | 13.57 | 3.72 |
| 9.42 | 3.16 | 2.05 | -5.82 | -4.85 | 10.02 | 10.93 | 0.63 | 4.88 | -1.73 | -10.12 | 10.32 | 7.00 | 2.88 | -10.65 | -6.94 | 12.01 | 13.35 | 2.03 |
| 8.83 | 2.70 | 2.63 | -5.66 | -5.76 | 9.23 | 10.41 | 0.52 | 5.45 | -1.32 | -9.68 | 10.31 | 6.76 | 2.30 | -10.42 | -7.20 | 12.46 | 13.34 | 0.90 |
| 7.28 | 2.28 | 3.61 | -5.00 | -5.64 | 8.37 | 9.67 | 1.09 | 6.32 | -0.50 | -8.65 | 9.94 | 6.78 | 2.23 | -9.64 | -6.52 | 12.75 | 13.25 | 0.52 |
| 5.76 | 2.20 | 4.47 | -4.06 | -4.84 | 7.50 | 9.17 | 1.99 | 7.08 | 0.28 | -7.41 | 9.31 | 7.17 | 2.64 | -8.59 | -5.14 | 12.61 | 13.02 | 0.69 |
| 4.93 | 2.53 | 4.72 | -3.22 | -4.05 | 6.65 | 8.84 | 2.54 | 7.21 | 0.56 | -6.53 | 8.48 | 7.69 | 3.16 | -7.59 | -3.53 | 12.22 | 12.58 | 0.96 |
| 4.78 | 3.16 | 4.27 | -2.86 | -3.82 | 6.00 | 8.26 | 2.41 | 6.54 | 0.19 | -6.30 | 7.76 | 7.80 | 3.30 | -6.97 | -2.05 | 11.92 | 11.83 | 1.08 |
| 5.34 | 3.84 | 3.57 | -2.81 | -3.83 | 5.82 | 7.43 | 2.00 | 5.55 | -0.50 | -6.47 | 7.66 | 7.31 | 2.99 | -6.61 | -0.66 | 11.98 | 10.72 | 1.29 |
| 6.44 | 4.62 | 3.32 | -2.62 | -3.57 | 6.10 | 6.89 | 2.01 | 4.95 | -0.93 | -6.47 | 8.38 | 6.53 | 2.54 | -6.20 | 0.97 | 12.41 | 9.57 | 2.01 |
| 7.49 | 5.59 | 3.78 | -1.89 | -2.85 | 6.57 | 6.83 | 2.68 | 5.28 | -0.68 | -6.01 | 9.23 | 6.03 | 2.18 | -5.42 | 2.77 | 12.99 | 8.91 | 3.56 |
| 8.03 | 6.86 | 4.85 | -0.53 | -2.01 | 6.99 | 7.22 | 3.82 | 6.57 | 0.26 | -5.17 | 9.36 | 6.27 | 2.08 | -4.40 | 4.22 | 13.62 | 9.18 | 5.50 |
| 8.13 | 8.41 | 6.01 | 1.12 | -1.40 | 7.37 | 7.87 | 5.07 | 8.51 | 1.60 | -4.34 | 8.68 | 7.19 | 2.30 | -3.41 | 5.04 | 14.20 | 10.43 | 7.24 |
| 8.38 | 9.92 | 7.06 | 2.77 | -0.94 | 7.83 | 8.61 | 6.27 | 10.77 | 3.01 | -3.53 | 7.90 | 8.59 | 3.11 | -2.49 | 5.47 | 14.62 | 12.22 | 8.62 |
| 9.00 | 11.08 | 8.00 | 4.22 | -0.19 | 8.16 | 9.41 | 7.33 | 12.88 | 4.52 | -2.72 | 7.98 | 10.39 | 4.56 | -1.49 | 6.09 | 14.96 | 13.89 | 9.77 |
| 9.87 | 11.93 | 8.74 | 5.43 | 0.83 | 8.00 | 10.02 | 8.18 | 14.52 | 5.89 | -1.83 | 9.36 | 12.32 | 6.35 | -0.52 | 6.97 | 15.19 | 15.10 | 10.73 |
| 10.68 | 12.69 | 9.24 | 6.30 | 1.90 | 7.39 | 10.30 | 8.87 | 15.49 | 6.88 | -0.96 | 11.47 | 14.07 | 7.92 | 0.20 | 7.67 | 15.36 | 15.87 | 11.28 |
| 11.12 | 13.30 | 9.48 | 6.67 | 2.55 | 6.77 | 10.67 | 9.36 | 15.81 | 7.26 | -0.49 | 12.97 | 15.16 | 8.77 | 0.46 | 7.70 | 15.46 | 16.52 | 11.28 |
| 11.15 | 13.31 | 9.54 | 6.59 | 2.53 | 6.75 | 11.61 | 9.66 | 15.61 | 6.99 | -0.48 | 12.91 | 15.34 | 8.76 | 0.33 | 7.17 | 15.65 | 17.07 | 11.11 |
| 10.95 | 12.41 | 9.50 | 6.42 | 2.03 | 7.45 | 12.84 | 9.95 | 14.88 | 6.32 | -0.87 | 11.66 | 14.89 | 8.19 | 0.18 | 6.61 | 16.07 | 17.46 | 11.12 |
| 10.86 | 10.83 | 9.24 | 6.28 | 1.07 | 8.47 | 13.43 | 10.21 | 13.62 | 5.42 | -1.56 | 10.59 | 14.13 | 7.16 | 0.15 | 6.23 | 16.61 | 17.72 | 11.30 |
| 11.31 | 9.26 | 8.79 | 6.41 | -0.14 | 9.18 | 12.98 | 10.66 | 12.19 | 4.49 | -2.31 | 10.74 | 13.41 | 5.75 | 0.22 | 5.96 | 17.04 | 18.17 | 11.34 |
| 12.46 | 8.48 | 8.49 | 6.96 | -0.87 | 9.61 | 12.46 | 11.39 | 11.25 | 4.11 | -2.65 | 11.70 | 13.02 | 4.41 | 0.46 | 5.89 | 17.32 | 18.96 | 11.19 |
| 13.90 | 8.66 | 8.72 | 7.92 | -1.25 | 10.17 | 12.89 | 12.43 | 11.29 | 4.54 | -2.14 | 12.82 | 13.25 | 3.16 | 1.02 | 6.12 | 17.50 | 19.56 | 10.97 |
| 14.90 | 9.25 | 9.31 | 8.85 | -2.35 | 10.93 | 14.06 | 13.39 | 11.84 | 5.45 | -1.00 | 13.59 | 13.84 | 1.38 | 1.61 | 6.30 | 17.39 | 19.30 | 10.48 |
| 14.39 | 9.27 | 9.59 | 8.82 | -4.08 | 11.47 | 14.71 | 13.74 | 11.96 | 5.92 | -0.09 | 13.55 | 13.91 | -0.78 | 1.66 | 5.79 | 16.71 | 17.86 | 9.40 |
| 12.29 | 8.32 | 8.91 | 7.10 | -5.32 | 11.10 | 14.04 | 12.92 | 11.04 | 5.35 | -0.22 | 12.44 | 12.96 | -2.24 | 0.79 | 4.37 | 15.49 | 15.70 | 7.56 |
| 9.74 | 6.89 | 7.42 | 4.01 | -4.79 | 9.94 | 12.53 | 11.20 | 9.54 | 4.06 | -1.37 | 10.54 | 11.32 | -1.84 | -0.57 | 2.80 | 14.01 | 13.63 | 5.75 |
| 7.88 | 5.73 | 5.82 | 0.82 | -2.54 | 8.71 | 10.91 | 9.44 | 8.41 | 3.02 | -2.69 | 8.84 | 10.03 | 0.39 | -1.38 | 2.13 | 12.72 | 12.37 | 5.05 |
| 7.02 | 4.96 | 4.59 | -1.47 | -0.21 | 7.66 | 9.29 | 8.07 | 7.96 | 2.56 | -3.46 | 8.07 | 9.62 | 2.92 | -1.08 | 2.67 | 11.74 | 12.04 | 5.73 |
| 6.51 | 4.27 | 3.66 | -2.82 | 1.30 | 6.53 | 7.51 | 7.00 | 7.70 | 2.45 | -3.65 | 7.81 | 9.65 | 4.70 | 0.05 | 3.84 | 11.18 | 12.20 | 7.21 |
| 5.85 | 3.36 | 2.89 | -3.61 | 2.18 | 5.15 | 5.98 | 5.91 | 7.31 | 2.30 | -3.53 | 7.19 | 9.44 | 5.79 | 1.38 | 4.96 | 11.09 | 12.44 | 8.83 |
| 4.93 | 2.13 | 2.32 | -4.04 | 2.56 | 3.79 | 5.25 | 4.83 | 6.63 | 2.11 | -3.28 | 6.05 | 8.62 | 6.47 | 2.56 | 5.82 | 11.04 | 12.40 | 10.10 |
| 3.83 | 0.91 | 2.09 | -4.02 | 2.72 | 2.73 | 5.44 | 4.05 | 5.93 | 2.11 | -2.83 | 4.97 | 7.36 | 6.91 | 3.51 | 6.37 | 10.55 | 12.09 | 10.87 |
| 2.97 | 0.07 | 2.10 | -3.38 | 2.93 | 2.16 | 6.07 | 3.87 | 5.41 | 2.35 | -2.05 | 4.69 | 6.12 | 7.11 | 4.36 | 6.69 | 9.58 | 11.77 | 11.01 |
| 2.82 | -0.19 | 2.04 | -2.16 | 3.32 | 2.10 | 6.52 | 4.24 | 4.99 | 2.54 | -1.37 | 5.24 | 5.28 | 6.99 | 4.96 | 6.72 | 8.92 | 11.56 | 10.77 |
| 3.21 | -0.11 | 1.65 | -0.76 | 3.93 | 2.51 | 6.32 | 4.90 | 4.60 | 2.33 | -1.01 | 6.20 | 4.97 | 6.73 | 5.29 | 6.77 | 9.19 | 11.55 | 10.72 |
| 3.68 | -0.07 | 0.89 | 0.35 | 4.64 | 2.93 | 5.31 | 5.70 | 4.23 | 1.66 | -0.94 | 6.85 | 4.98 | 6.41 | 5.39 | 7.09 | 10.09 | 11.40 | 11.21 |

Each cell corresponds to a time instant starting from 5 ms to 1000 ms (y-axis) in every scalp electrode site (x-axis) where the EEG signal was recorded. Averaged-voltage values are in microvolts (μV).

**Table 3. Grand-mean voltages in EEG epochs with correct responses in children with low mathematical achievement level (n=20).**

| **Fp2** | **F4** | **C4** | **P4** | **O2** | **F8** | **T4** | **T6** | **Fz** | **Cz** | **Pz** | **Fp1** | **F3** | **C3** | **P3** | **O1** | **F7** | **T3** | **T5** |
| --- | --- | --- | --- | --- | --- | --- | --- | --- | --- | --- | --- | --- | --- | --- | --- | --- | --- | --- |
| 10.61 | 6.97 | 4.65 | 6.32 | 9.79 | 17.18 | 4.92 | 5.92 | 8.16 | 4.86 | 6.38 | 10.86 | 10.49 | 8.38 | 8.49 | 9.06 | 12.97 | 11.60 | 8.51 |
| 9.24 | 8.13 | 3.89 | 5.71 | 8.27 | 15.86 | 5.04 | 4.82 | 7.72 | 4.81 | 5.45 | 10.20 | 10.07 | 9.80 | 8.22 | 8.09 | 12.71 | 11.81 | 9.68 |
| 8.90 | 9.28 | 3.56 | 5.20 | 6.98 | 14.96 | 4.92 | 4.27 | 7.55 | 5.17 | 5.12 | 10.91 | 10.10 | 11.21 | 8.34 | 7.06 | 13.59 | 12.14 | 10.89 |
| 10.03 | 10.23 | 3.95 | 4.85 | 6.15 | 14.99 | 4.39 | 4.10 | 7.92 | 5.99 | 5.34 | 12.79 | 10.69 | 12.27 | 8.70 | 6.20 | 15.34 | 13.20 | 11.64 |
| 11.72 | 10.75 | 4.77 | 4.82 | 5.25 | 15.74 | 4.10 | 3.97 | 8.64 | 7.04 | 5.64 | 15.08 | 11.34 | 12.64 | 8.85 | 5.23 | 17.09 | 14.93 | 11.27 |
| 12.51 | 10.84 | 5.66 | 5.14 | 3.92 | 16.56 | 4.81 | 3.95 | 9.37 | 7.99 | 5.77 | 16.51 | 11.45 | 12.38 | 8.60 | 3.88 | 18.03 | 16.24 | 9.91 |
| 12.09 | 10.90 | 6.73 | 5.88 | 2.43 | 17.09 | 6.62 | 4.39 | 10.21 | 8.91 | 5.96 | 16.62 | 11.18 | 12.07 | 8.21 | 2.38 | 18.10 | 16.34 | 8.43 |
| 11.57 | 11.18 | 8.30 | 6.92 | 1.80 | 17.75 | 8.78 | 5.65 | 11.38 | 10.15 | 6.82 | 16.06 | 11.43 | 12.35 | 8.29 | 1.58 | 18.11 | 15.62 | 7.85 |
| 12.23 | 11.61 | 10.33 | 7.98 | 2.59 | 18.71 | 10.47 | 7.35 | 12.87 | 11.77 | 8.43 | 15.94 | 12.62 | 13.37 | 8.95 | 1.87 | 18.64 | 15.01 | 8.22 |
| 13.88 | 11.95 | 12.21 | 8.75 | 4.29 | 19.55 | 11.26 | 8.60 | 14.19 | 13.26 | 10.08 | 16.63 | 14.13 | 14.57 | 9.92 | 3.07 | 19.43 | 15.13 | 8.90 |
| 15.52 | 12.36 | 13.14 | 9.04 | 5.95 | 19.93 | 11.42 | 8.91 | 14.76 | 13.96 | 10.86 | 17.52 | 15.19 | 15.10 | 10.67 | 4.68 | 19.89 | 15.87 | 9.34 |
| 16.45 | 12.94 | 13.09 | 9.02 | 7.14 | 20.05 | 11.36 | 8.46 | 14.50 | 13.70 | 10.45 | 18.05 | 15.39 | 14.46 | 11.02 | 6.15 | 19.52 | 16.39 | 9.45 |
| 16.59 | 13.42 | 12.45 | 8.87 | 8.30 | 20.41 | 11.45 | 8.17 | 13.66 | 12.75 | 9.53 | 18.01 | 14.67 | 12.84 | 11.04 | 7.43 | 18.25 | 15.96 | 9.44 |
| 16.24 | 13.52 | 11.83 | 8.87 | 9.89 | 20.86 | 11.60 | 8.67 | 12.62 | 11.49 | 8.93 | 17.50 | 13.37 | 10.80 | 10.69 | 8.72 | 16.37 | 14.68 | 9.41 |
| 15.44 | 13.11 | 11.44 | 8.88 | 11.83 | 20.94 | 11.86 | 9.91 | 11.54 | 10.18 | 8.87 | 16.62 | 11.54 | 8.87 | 9.95 | 10.12 | 14.33 | 13.00 | 9.41 |
| 14.39 | 12.42 | 11.13 | 8.83 | 13.52 | 20.52 | 12.62 | 11.38 | 10.46 | 8.88 | 9.18 | 15.43 | 9.64 | 7.27 | 8.89 | 11.34 | 12.58 | 11.37 | 9.30 |
| 13.57 | 11.51 | 10.78 | 8.52 | 14.44 | 19.95 | 13.62 | 12.68 | 9.39 | 7.73 | 9.38 | 14.02 | 8.11 | 6.06 | 7.87 | 12.11 | 11.37 | 10.02 | 9.05 |
| 13.31 | 10.33 | 10.49 | 7.85 | 14.45 | 19.43 | 13.57 | 13.68 | 8.52 | 6.90 | 9.53 | 12.67 | 7.21 | 5.32 | 7.28 | 12.40 | 10.84 | 8.95 | 8.87 |
| 13.59 | 9.19 | 10.46 | 6.76 | 13.64 | 19.02 | 11.70 | 14.02 | 8.08 | 6.61 | 9.55 | 11.76 | 7.21 | 5.17 | 7.08 | 12.25 | 10.96 | 8.44 | 8.85 |
| 14.03 | 8.78 | 10.86 | 5.63 | 12.29 | 18.81 | 9.21 | 13.91 | 8.32 | 6.93 | 9.31 | 11.75 | 8.28 | 5.55 | 7.05 | 11.66 | 11.44 | 8.75 | 9.02 |
| 14.39 | 9.45 | 11.71 | 5.14 | 11.21 | 18.81 | 8.11 | 13.82 | 9.15 | 7.75 | 8.96 | 12.56 | 9.93 | 6.36 | 7.05 | 10.62 | 11.86 | 9.74 | 9.28 |
| 14.56 | 10.96 | 12.69 | 5.51 | 10.98 | 19.04 | 9.18 | 14.11 | 10.01 | 8.51 | 8.52 | 13.45 | 11.13 | 7.23 | 6.96 | 9.23 | 11.84 | 11.00 | 9.42 |
| 14.57 | 12.39 | 13.25 | 6.33 | 11.36 | 19.26 | 11.02 | 14.55 | 10.15 | 8.48 | 7.86 | 13.89 | 10.88 | 7.53 | 6.62 | 7.60 | 11.41 | 11.59 | 9.15 |
| 14.63 | 13.13 | 13.00 | 6.97 | 11.60 | 19.33 | 12.18 | 14.76 | 9.39 | 7.25 | 6.89 | 13.65 | 9.10 | 6.87 | 5.83 | 5.87 | 10.87 | 11.29 | 8.28 |
| 14.60 | 13.09 | 12.07 | 7.26 | 11.45 | 19.12 | 12.36 | 14.75 | 8.16 | 5.41 | 5.87 | 12.78 | 6.86 | 5.66 | 4.92 | 4.54 | 10.37 | 10.23 | 7.31 |
| 14.29 | 12.78 | 11.00 | 7.56 | 11.47 | 18.62 | 11.92 | 14.97 | 7.38 | 3.87 | 5.31 | 11.58 | 5.49 | 4.68 | 4.43 | 4.10 | 10.08 | 9.34 | 6.76 |
| 13.79 | 12.66 | 10.21 | 8.14 | 11.99 | 17.96 | 11.13 | 15.33 | 7.37 | 3.20 | 5.33 | 10.55 | 5.45 | 4.40 | 4.60 | 4.63 | 9.96 | 9.06 | 6.77 |
| 13.29 | 12.67 | 9.73 | 8.68 | 12.78 | 17.28 | 10.18 | 15.49 | 7.88 | 3.28 | 5.61 | 10.05 | 6.19 | 4.83 | 5.28 | 5.78 | 9.82 | 8.64 | 6.95 |
| 13.24 | 12.47 | 9.38 | 8.85 | 13.25 | 16.84 | 9.28 | 15.24 | 8.44 | 3.76 | 5.93 | 10.18 | 6.89 | 5.60 | 6.18 | 7.13 | 9.62 | 7.51 | 7.06 |
| 13.46 | 11.93 | 8.78 | 8.51 | 13.02 | 16.44 | 8.99 | 14.50 | 8.72 | 4.29 | 6.08 | 10.48 | 7.17 | 6.36 | 7.02 | 8.28 | 9.45 | 5.95 | 7.09 |
| 13.53 | 11.24 | 7.84 | 7.74 | 12.03 | 16.06 | 9.52 | 13.31 | 8.81 | 4.60 | 6.13 | 10.28 | 7.32 | 6.77 | 7.75 | 9.09 | 9.66 | 4.94 | 7.26 |
| 13.30 | 10.81 | 6.93 | 6.74 | 10.69 | 15.82 | 10.16 | 12.02 | 9.06 | 4.82 | 6.11 | 9.67 | 7.80 | 6.89 | 8.32 | 9.54 | 10.36 | 5.05 | 7.59 |
| 12.97 | 10.96 | 6.56 | 5.88 | 9.32 | 15.75 | 9.99 | 11.16 | 9.80 | 5.07 | 6.38 | 9.42 | 8.48 | 7.01 | 8.87 | 9.98 | 11.43 | 6.03 | 8.34 |
| 12.93 | 11.63 | 6.89 | 5.23 | 8.16 | 15.58 | 8.72 | 10.84 | 10.95 | 5.61 | 7.12 | 10.16 | 9.08 | 7.29 | 9.48 | 10.62 | 12.46 | 7.53 | 9.51 |
| 13.12 | 12.37 | 7.74 | 4.78 | 7.54 | 15.05 | 7.02 | 10.78 | 12.03 | 6.30 | 8.08 | 11.47 | 9.42 | 7.67 | 10.02 | 11.32 | 13.19 | 9.25 | 10.86 |
| 13.36 | 13.04 | 8.69 | 4.28 | 7.63 | 14.30 | 5.94 | 10.91 | 12.56 | 6.90 | 9.03 | 12.29 | 9.51 | 7.89 | 10.12 | 11.74 | 13.57 | 10.87 | 12.02 |
| 13.66 | 13.73 | 9.65 | 3.73 | 8.42 | 13.99 | 6.07 | 11.44 | 12.75 | 7.41 | 9.81 | 12.15 | 9.54 | 8.06 | 9.67 | 11.60 | 13.73 | 11.64 | 12.64 |
| 13.93 | 14.50 | 10.63 | 3.37 | 9.54 | 14.32 | 7.35 | 12.38 | 13.07 | 7.90 | 10.38 | 11.65 | 9.81 | 8.38 | 8.85 | 11.05 | 13.99 | 11.51 | 12.48 |
| 14.08 | 14.96 | 11.56 | 3.36 | 10.49 | 14.97 | 9.11 | 13.38 | 13.57 | 8.48 | 10.60 | 11.46 | 10.72 | 9.01 | 7.92 | 10.35 | 14.63 | 11.29 | 11.70 |
| 14.02 | 14.66 | 12.21 | 3.48 | 10.96 | 15.36 | 10.41 | 13.97 | 13.84 | 9.06 | 10.49 | 11.95 | 11.99 | 9.94 | 7.23 | 9.57 | 15.58 | 11.55 | 10.77 |
| 13.90 | 13.77 | 12.38 | 3.43 | 10.94 | 15.32 | 10.71 | 14.11 | 13.59 | 9.54 | 10.36 | 12.89 | 12.84 | 10.88 | 6.90 | 8.62 | 16.57 | 12.07 | 10.19 |
| 13.84 | 13.07 | 12.23 | 3.32 | 10.52 | 15.15 | 10.24 | 13.91 | 12.96 | 9.86 | 10.39 | 13.83 | 12.74 | 11.45 | 6.75 | 7.43 | 17.08 | 12.04 | 10.07 |
| 13.91 | 12.91 | 12.09 | 3.59 | 10.00 | 15.25 | 10.05 | 13.58 | 12.40 | 10.00 | 10.59 | 14.48 | 11.86 | 11.46 | 6.63 | 6.27 | 17.12 | 11.42 | 10.27 |
| 13.98 | 12.90 | 12.19 | 4.38 | 9.82 | 15.71 | 10.95 | 13.31 | 12.12 | 9.90 | 10.79 | 14.58 | 10.75 | 11.02 | 6.67 | 5.60 | 16.63 | 11.43 | 10.71 |
| 13.97 | 12.62 | 12.40 | 5.40 | 10.18 | 16.04 | 12.63 | 13.13 | 11.90 | 9.58 | 10.78 | 13.74 | 9.66 | 10.15 | 6.96 | 5.66 | 15.52 | 12.35 | 11.29 |
| 13.88 | 12.05 | 12.43 | 5.95 | 10.82 | 15.89 | 14.11 | 12.62 | 11.43 | 8.98 | 10.37 | 11.82 | 8.55 | 8.90 | 7.37 | 6.26 | 13.68 | 13.29 | 11.57 |
| 13.67 | 11.69 | 11.95 | 5.77 | 11.21 | 15.39 | 14.42 | 11.48 | 10.73 | 8.20 | 9.62 | 9.48 | 7.42 | 7.30 | 7.66 | 7.18 | 11.93 | 13.63 | 11.16 |
| 13.46 | 11.82 | 11.11 | 5.17 | 11.24 | 14.93 | 13.35 | 10.12 | 10.11 | 7.34 | 8.61 | 8.13 | 6.68 | 5.70 | 7.81 | 8.27 | 11.16 | 13.71 | 10.47 |
| 13.39 | 12.30 | 10.18 | 4.72 | 11.33 | 14.90 | 11.63 | 9.09 | 9.81 | 6.69 | 7.92 | 8.60 | 6.59 | 4.50 | 8.08 | 9.58 | 11.74 | 13.79 | 10.01 |
| 13.45 | 12.53 | 9.28 | 4.74 | 11.76 | 15.39 | 9.97 | 8.66 | 9.66 | 6.22 | 7.67 | 9.92 | 6.83 | 3.76 | 8.54 | 10.93 | 12.77 | 12.77 | 10.10 |
| 13.32 | 12.05 | 8.40 | 4.94 | 12.23 | 15.84 | 8.94 | 8.49 | 9.26 | 5.64 | 7.55 | 10.57 | 6.72 | 3.21 | 8.86 | 12.18 | 13.03 | 10.12 | 10.31 |
| 12.64 | 10.81 | 7.34 | 4.81 | 12.48 | 15.60 | 8.60 | 8.20 | 8.42 | 4.85 | 7.20 | 10.10 | 5.87 | 2.70 | 8.73 | 13.24 | 12.21 | 7.04 | 10.33 |
| 11.53 | 9.27 | 6.29 | 4.30 | 12.46 | 14.55 | 8.48 | 7.70 | 7.51 | 4.12 | 6.70 | 9.17 | 4.73 | 2.37 | 8.40 | 14.17 | 10.78 | 5.62 | 10.11 |
| 10.82 | 7.95 | 5.55 | 3.81 | 12.51 | 13.39 | 8.55 | 6.91 | 6.97 | 3.89 | 6.51 | 8.85 | 4.11 | 2.55 | 8.26 | 14.99 | 9.93 | 6.21 | 9.92 |
| 11.36 | 6.92 | 5.16 | 3.76 | 12.67 | 12.62 | 8.97 | 5.90 | 6.98 | 4.31 | 6.90 | 9.57 | 4.32 | 3.41 | 8.61 | 15.70 | 10.46 | 7.55 | 9.62 |
| 12.83 | 6.20 | 4.97 | 3.99 | 12.60 | 12.33 | 9.38 | 4.82 | 7.38 | 5.18 | 7.74 | 10.71 | 5.06 | 4.59 | 9.19 | 16.07 | 12.24 | 8.77 | 9.16 |
| 14.26 | 6.00 | 4.94 | 4.05 | 12.05 | 12.25 | 9.24 | 3.84 | 7.99 | 6.32 | 8.72 | 11.41 | 5.79 | 5.62 | 9.61 | 15.71 | 14.39 | 9.86 | 8.60 |
| 15.05 | 6.67 | 5.12 | 3.95 | 11.03 | 12.20 | 7.97 | 3.20 | 8.94 | 7.75 | 9.64 | 11.28 | 6.53 | 6.45 | 9.74 | 14.45 | 16.21 | 11.07 | 8.18 |
| 15.27 | 8.16 | 5.63 | 4.09 | 9.90 | 12.32 | 5.87 | 2.92 | 10.25 | 9.51 | 10.41 | 11.13 | 7.59 | 7.29 | 9.63 | 12.60 | 17.46 | 11.94 | 7.78 |
| 15.28 | 10.04 | 6.42 | 4.73 | 9.02 | 12.73 | 4.08 | 3.11 | 11.66 | 11.20 | 11.10 | 11.95 | 8.90 | 8.24 | 9.61 | 10.66 | 18.07 | 11.84 | 7.13 |
| 15.38 | 11.55 | 7.30 | 5.78 | 8.43 | 13.27 | 3.36 | 3.86 | 12.66 | 12.47 | 11.73 | 14.03 | 10.12 | 9.08 | 9.80 | 8.89 | 18.08 | 11.36 | 6.01 |
| 15.75 | 12.38 | 8.21 | 7.04 | 7.82 | 13.98 | 4.09 | 5.12 | 13.25 | 13.35 | 12.38 | 16.50 | 11.03 | 9.86 | 10.24 | 7.30 | 17.86 | 11.62 | 5.11 |
| 16.17 | 12.81 | 9.28 | 8.17 | 7.19 | 14.50 | 5.96 | 6.67 | 13.81 | 14.17 | 13.16 | 17.95 | 11.67 | 10.93 | 10.93 | 5.93 | 17.87 | 12.79 | 5.28 |
| 16.30 | 13.46 | 10.71 | 9.08 | 6.78 | 14.67 | 8.05 | 8.37 | 14.66 | 15.22 | 14.21 | 17.66 | 12.41 | 12.52 | 12.07 | 5.27 | 18.37 | 14.28 | 6.90 |
| 15.97 | 14.71 | 12.56 | 9.96 | 7.14 | 14.47 | 9.82 | 10.28 | 15.78 | 16.53 | 15.57 | 16.19 | 13.44 | 14.40 | 13.83 | 5.63 | 18.90 | 15.14 | 9.23 |
| 15.65 | 16.25 | 14.44 | 11.08 | 8.49 | 14.29 | 11.35 | 12.51 | 16.78 | 17.74 | 17.16 | 14.97 | 14.43 | 15.98 | 16.08 | 7.20 | 19.01 | 15.14 | 11.16 |
| 15.57 | 17.36 | 15.85 | 12.33 | 10.36 | 14.22 | 12.95 | 14.72 | 17.22 | 18.46 | 18.55 | 15.06 | 14.90 | 16.80 | 18.27 | 9.63 | 18.63 | 14.81 | 12.03 |
| 15.66 | 17.79 | 16.54 | 13.28 | 12.11 | 14.49 | 14.56 | 16.42 | 17.01 | 18.68 | 19.30 | 16.40 | 14.53 | 16.99 | 19.86 | 12.29 | 18.19 | 14.80 | 12.31 |
| 15.96 | 18.05 | 16.84 | 13.59 | 13.51 | 15.35 | 15.52 | 17.43 | 16.60 | 18.73 | 19.32 | 17.92 | 13.70 | 17.05 | 20.89 | 14.68 | 18.28 | 15.22 | 12.88 |
| 16.41 | 18.49 | 17.32 | 13.50 | 14.65 | 16.70 | 15.73 | 18.05 | 16.48 | 18.94 | 18.77 | 18.84 | 13.17 | 17.37 | 21.63 | 16.58 | 18.99 | 15.66 | 14.23 |
| 16.65 | 18.98 | 18.15 | 13.51 | 15.90 | 17.81 | 16.33 | 18.78 | 16.83 | 19.35 | 18.00 | 18.91 | 13.41 | 18.10 | 22.40 | 18.24 | 19.90 | 16.02 | 16.03 |
| 16.31 | 18.92 | 18.86 | 13.68 | 17.15 | 17.98 | 17.48 | 19.68 | 17.31 | 19.59 | 16.99 | 18.33 | 14.29 | 18.89 | 23.08 | 19.73 | 20.41 | 16.87 | 17.41 |
| 15.33 | 18.01 | 18.99 | 13.92 | 18.18 | 17.00 | 18.05 | 20.33 | 17.53 | 19.44 | 16.04 | 17.19 | 15.55 | 19.37 | 23.43 | 20.78 | 20.16 | 18.54 | 17.81 |
| 14.22 | 16.57 | 18.48 | 14.12 | 18.64 | 15.70 | 17.29 | 20.32 | 17.39 | 18.90 | 15.47 | 15.89 | 16.78 | 19.38 | 23.39 | 20.93 | 19.33 | 20.02 | 17.47 |
| 13.15 | 14.79 | 17.58 | 14.22 | 18.39 | 14.63 | 15.66 | 19.55 | 16.81 | 18.13 | 15.43 | 14.74 | 17.46 | 19.11 | 23.18 | 20.11 | 18.64 | 19.85 | 17.01 |
| 12.12 | 12.96 | 16.54 | 14.04 | 17.50 | 13.99 | 14.56 | 18.28 | 15.61 | 17.05 | 15.46 | 14.05 | 17.07 | 18.58 | 22.80 | 18.54 | 18.55 | 18.13 | 16.78 |
| 11.47 | 11.60 | 15.56 | 13.22 | 16.22 | 13.64 | 14.35 | 16.91 | 13.99 | 15.69 | 14.97 | 14.12 | 15.57 | 17.66 | 22.12 | 16.48 | 18.77 | 16.44 | 16.47 |
| 11.80 | 11.25 | 14.72 | 11.77 | 14.73 | 13.67 | 14.65 | 15.76 | 12.41 | 14.21 | 14.02 | 14.71 | 13.64 | 16.29 | 20.88 | 14.13 | 18.55 | 15.61 | 15.54 |
| 13.21 | 11.98 | 13.94 | 9.95 | 13.04 | 14.22 | 15.01 | 14.63 | 11.61 | 12.91 | 13.01 | 15.51 | 11.94 | 14.62 | 19.11 | 11.66 | 17.81 | 14.95 | 13.96 |
| 15.06 | 13.21 | 13.05 | 8.32 | 10.97 | 14.93 | 15.08 | 13.37 | 11.78 | 11.97 | 12.33 | 16.51 | 10.83 | 13.06 | 17.20 | 9.24 | 16.91 | 13.47 | 12.21 |
| 16.32 | 13.97 | 11.81 | 7.23 | 8.64 | 15.17 | 14.36 | 11.87 | 12.61 | 11.46 | 11.96 | 17.46 | 10.51 | 11.95 | 15.55 | 7.04 | 16.53 | 11.90 | 10.74 |
| 16.24 | 13.63 | 10.34 | 6.95 | 6.83 | 14.52 | 13.17 | 10.47 | 13.54 | 11.17 | 11.79 | 17.93 | 10.97 | 11.49 | 14.44 | 5.28 | 16.74 | 11.62 | 10.03 |
| 15.15 | 12.49 | 8.90 | 7.18 | 6.03 | 13.18 | 12.84 | 10.00 | 14.02 | 10.98 | 11.84 | 17.58 | 11.82 | 11.53 | 13.95 | 4.29 | 17.11 | 13.03 | 10.17 |
| 13.88 | 11.25 | 7.89 | 7.37 | 6.03 | 11.99 | 13.67 | 10.64 | 13.88 | 10.67 | 12.01 | 16.42 | 12.60 | 11.82 | 13.86 | 3.90 | 17.13 | 14.54 | 11.03 |
| 13.35 | 10.56 | 7.52 | 7.02 | 6.02 | 11.49 | 14.71 | 11.68 | 13.51 | 10.28 | 12.01 | 15.05 | 12.97 | 12.01 | 13.78 | 3.69 | 16.80 | 14.45 | 11.96 |
| 13.44 | 10.73 | 7.63 | 6.08 | 5.24 | 11.91 | 14.78 | 12.20 | 13.26 | 9.88 | 11.65 | 14.03 | 12.97 | 12.03 | 13.44 | 3.17 | 16.40 | 12.84 | 12.19 |
| 13.59 | 11.51 | 7.82 | 5.01 | 3.91 | 13.01 | 13.68 | 11.75 | 13.14 | 9.39 | 10.77 | 13.33 | 12.85 | 11.71 | 12.94 | 2.51 | 16.00 | 11.07 | 11.40 |
| 13.61 | 12.44 | 7.72 | 4.35 | 2.80 | 14.35 | 12.59 | 10.80 | 12.98 | 8.78 | 9.58 | 12.63 | 12.64 | 11.17 | 12.53 | 2.06 | 15.32 | 10.29 | 10.17 |
| 14.03 | 13.37 | 7.41 | 4.26 | 2.63 | 15.77 | 12.75 | 10.01 | 12.82 | 8.25 | 8.58 | 12.16 | 12.44 | 10.74 | 12.48 | 2.34 | 14.27 | 10.25 | 9.43 |
| 14.99 | 14.19 | 7.24 | 4.61 | 3.62 | 17.08 | 14.35 | 9.46 | 12.73 | 8.12 | 8.15 | 12.24 | 12.22 | 10.70 | 12.81 | 3.38 | 13.28 | 10.10 | 9.72 |
| 15.93 | 14.72 | 7.35 | 5.15 | 5.41 | 17.82 | 16.49 | 8.91 | 12.66 | 8.24 | 8.41 | 12.87 | 11.91 | 10.98 | 13.22 | 5.00 | 13.00 | 9.54 | 10.54 |
| 16.21 | 14.54 | 7.41 | 5.50 | 7.45 | 17.54 | 17.92 | 8.34 | 12.24 | 8.15 | 9.05 | 13.59 | 11.08 | 11.06 | 13.29 | 6.81 | 13.23 | 9.04 | 11.16 |
| 15.56 | 13.66 | 7.02 | 5.39 | 9.04 | 16.14 | 18.04 | 7.75 | 11.19 | 7.57 | 9.53 | 13.64 | 9.53 | 10.49 | 12.65 | 8.28 | 13.36 | 9.21 | 11.02 |
| 14.30 | 12.68 | 6.14 | 4.85 | 9.67 | 14.56 | 17.08 | 7.04 | 9.92 | 6.87 | 9.50 | 12.87 | 7.94 | 9.42 | 11.58 | 8.83 | 12.79 | 9.93 | 10.21 |
| 13.08 | 12.38 | 5.04 | 4.13 | 9.26 | 13.66 | 15.50 | 5.90 | 9.04 | 6.57 | 9.17 | 11.76 | 7.37 | 8.59 | 10.48 | 8.34 | 11.73 | 10.57 | 9.12 |
| 12.35 | 12.83 | 4.18 | 3.48 | 8.18 | 13.71 | 13.67 | 4.55 | 8.90 | 6.95 | 9.00 | 10.90 | 8.20 | 8.51 | 9.85 | 7.42 | 10.76 | 10.45 | 8.16 |
| 12.49 | 13.65 | 3.92 | 2.96 | 7.10 | 14.12 | 11.99 | 3.53 | 9.39 | 7.90 | 9.42 | 10.65 | 9.78 | 9.10 | 9.84 | 6.79 | 10.51 | 9.63 | 7.61 |
| 13.29 | 14.35 | 4.16 | 2.49 | 6.38 | 14.42 | 11.16 | 3.38 | 10.08 | 9.05 | 10.35 | 11.19 | 11.16 | 9.73 | 10.25 | 6.67 | 11.06 | 8.82 | 7.37 |
| 14.17 | 14.66 | 4.62 | 2.02 | 5.95 | 14.55 | 11.63 | 3.79 | 10.64 | 10.16 | 11.28 | 12.43 | 11.78 | 9.93 | 10.72 | 6.62 | 12.03 | 8.66 | 6.92 |
| 14.78 | 14.39 | 4.98 | 1.48 | 5.37 | 14.72 | 13.40 | 3.89 | 11.06 | 11.10 | 11.81 | 13.83 | 11.39 | 9.72 | 10.96 | 5.95 | 12.83 | 9.19 | 5.99 |
| 14.94 | 13.50 | 5.31 | 1.20 | 4.65 | 14.68 | 15.74 | 3.44 | 11.32 | 11.91 | 12.04 | 14.39 | 10.32 | 9.44 | 10.94 | 4.72 | 13.08 | 9.91 | 4.74 |
| 14.58 | 12.28 | 5.79 | 1.35 | 4.11 | 13.98 | 17.24 | 2.94 | 11.49 | 12.44 | 12.21 | 13.68 | 9.15 | 9.35 | 10.78 | 3.50 | 12.71 | 10.58 | 3.93 |
| 13.82 | 11.41 | 6.35 | 1.75 | 4.01 | 12.77 | 16.83 | 2.85 | 11.66 | 12.57 | 12.37 | 12.41 | 8.31 | 9.37 | 10.42 | 2.73 | 12.04 | 11.14 | 3.87 |
| 12.89 | 11.47 | 6.92 | 2.12 | 4.33 | 11.77 | 14.72 | 3.14 | 11.78 | 12.28 | 12.28 | 11.77 | 7.97 | 9.27 | 9.86 | 2.42 | 11.26 | 11.32 | 4.29 |
| 11.97 | 12.65 | 7.45 | 2.45 | 5.20 | 11.44 | 12.47 | 3.77 | 11.68 | 11.70 | 11.79 | 11.94 | 7.87 | 9.04 | 9.21 | 2.61 | 10.45 | 10.97 | 5.06 |
| 11.30 | 14.14 | 7.86 | 3.01 | 6.72 | 11.66 | 11.44 | 4.75 | 11.24 | 11.14 | 11.04 | 12.24 | 7.91 | 8.76 | 8.79 | 3.27 | 9.73 | 10.15 | 5.99 |
| 11.05 | 14.73 | 8.06 | 4.31 | 8.87 | 12.02 | 11.43 | 6.33 | 10.50 | 10.64 | 10.37 | 12.02 | 8.16 | 8.55 | 8.77 | 4.60 | 9.30 | 9.35 | 7.10 |
| 11.26 | 13.88 | 7.96 | 6.42 | 11.45 | 12.08 | 11.19 | 8.31 | 9.61 | 10.22 | 10.06 | 11.00 | 8.46 | 8.43 | 9.23 | 6.64 | 9.16 | 9.02 | 8.44 |
| 11.51 | 12.06 | 7.70 | 8.89 | 13.91 | 11.51 | 10.34 | 10.18 | 8.81 | 9.78 | 10.07 | 9.36 | 8.77 | 8.35 | 10.00 | 9.27 | 9.23 | 9.40 | 10.01 |
| 11.32 | 10.13 | 7.47 | 10.79 | 16.03 | 10.46 | 9.74 | 11.59 | 8.32 | 9.25 | 10.25 | 7.70 | 8.88 | 8.33 | 10.68 | 11.93 | 9.10 | 10.04 | 11.60 |
| 10.53 | 8.50 | 7.35 | 11.46 | 17.61 | 9.42 | 9.94 | 12.42 | 7.98 | 8.39 | 10.11 | 6.52 | 8.53 | 8.00 | 10.83 | 13.84 | 8.51 | 10.23 | 12.62 |
| 9.57 | 7.21 | 7.22 | 11.16 | 18.74 | 9.03 | 10.71 | 12.81 | 7.40 | 6.96 | 9.53 | 6.07 | 7.54 | 7.08 | 10.21 | 14.66 | 7.54 | 9.35 | 12.71 |
| 8.94 | 6.27 | 7.12 | 10.76 | 19.69 | 9.55 | 11.63 | 13.03 | 6.51 | 5.16 | 8.75 | 6.37 | 6.00 | 5.67 | 9.05 | 14.72 | 6.52 | 7.68 | 11.94 |
| 9.21 | 5.79 | 7.10 | 10.79 | 20.41 | 10.69 | 12.33 | 13.23 | 5.53 | 3.53 | 8.23 | 7.43 | 4.51 | 4.25 | 7.90 | 14.57 | 5.86 | 6.15 | 10.74 |
| 10.34 | 5.64 | 6.98 | 11.17 | 20.42 | 11.76 | 12.55 | 13.06 | 4.79 | 2.48 | 8.19 | 8.99 | 3.46 | 3.30 | 7.10 | 14.30 | 5.77 | 4.88 | 9.54 |
| 11.52 | 5.51 | 6.65 | 11.51 | 19.83 | 12.02 | 11.88 | 12.46 | 4.51 | 2.22 | 8.68 | 9.95 | 3.12 | 2.98 | 6.86 | 13.99 | 6.13 | 3.81 | 8.75 |
| 11.96 | 5.41 | 6.05 | 11.90 | 19.28 | 11.26 | 10.52 | 11.77 | 4.59 | 2.64 | 9.67 | 9.50 | 3.58 | 3.27 | 7.28 | 13.99 | 6.97 | 3.33 | 8.84 |
| 11.67 | 5.72 | 5.42 | 12.47 | 19.00 | 9.99 | 9.13 | 11.46 | 4.64 | 3.50 | 11.02 | 7.96 | 4.42 | 3.90 | 8.22 | 14.43 | 8.02 | 4.02 | 9.89 |
| 11.05 | 6.49 | 4.99 | 13.25 | 18.76 | 8.79 | 7.95 | 12.01 | 4.41 | 4.52 | 12.43 | 6.48 | 4.77 | 4.68 | 9.30 | 15.10 | 8.52 | 5.44 | 11.35 |
| 10.21 | 6.97 | 4.96 | 13.98 | 18.05 | 7.87 | 6.83 | 13.23 | 3.89 | 5.59 | 13.75 | 5.74 | 4.13 | 5.54 | 10.19 | 15.59 | 7.78 | 6.39 | 12.56 |
| 9.44 | 6.59 | 5.41 | 14.55 | 16.87 | 7.16 | 6.39 | 14.66 | 3.42 | 6.74 | 15.01 | 6.05 | 3.01 | 6.62 | 10.92 | 15.66 | 6.00 | 6.18 | 13.33 |
| 9.25 | 5.65 | 6.11 | 15.14 | 15.55 | 6.87 | 7.69 | 15.86 | 3.42 | 8.16 | 16.41 | 7.20 | 2.51 | 8.12 | 11.59 | 15.12 | 4.18 | 5.34 | 13.96 |
| 10.14 | 4.94 | 6.69 | 15.73 | 14.43 | 7.07 | 10.60 | 16.54 | 3.89 | 9.58 | 17.60 | 8.58 | 2.97 | 9.69 | 12.04 | 13.90 | 3.32 | 4.41 | 14.62 |
| 11.56 | 4.82 | 6.88 | 15.89 | 13.76 | 7.50 | 13.27 | 16.60 | 4.65 | 10.62 | 17.97 | 9.31 | 3.73 | 10.75 | 12.05 | 12.29 | 3.39 | 3.57 | 15.14 |
| 12.29 | 5.10 | 6.57 | 15.36 | 13.56 | 8.02 | 13.87 | 16.24 | 5.34 | 11.20 | 17.20 | 9.06 | 4.12 | 10.92 | 11.66 | 10.92 | 3.76 | 3.23 | 15.14 |
| 11.69 | 5.51 | 6.20 | 14.33 | 13.65 | 8.73 | 12.75 | 15.74 | 6.02 | 11.62 | 15.65 | 8.22 | 4.21 | 10.50 | 11.14 | 10.17 | 3.92 | 4.03 | 14.72 |
| 10.28 | 6.07 | 6.10 | 13.03 | 13.30 | 9.52 | 11.37 | 15.17 | 6.77 | 12.27 | 13.87 | 7.33 | 4.42 | 9.89 | 10.56 | 9.89 | 3.85 | 5.88 | 14.11 |
| 9.16 | 6.82 | 6.22 | 11.31 | 12.17 | 9.97 | 10.81 | 14.55 | 7.50 | 13.15 | 12.02 | 6.49 | 4.98 | 9.31 | 9.71 | 9.45 | 3.69 | 7.48 | 13.34 |
| 8.85 | 7.56 | 6.24 | 8.84 | 10.45 | 9.70 | 11.00 | 13.50 | 7.99 | 13.79 | 9.99 | 5.71 | 5.62 | 8.64 | 8.33 | 8.38 | 3.61 | 7.86 | 12.25 |
| 9.09 | 8.06 | 5.72 | 5.73 | 8.54 | 9.19 | 11.23 | 11.75 | 8.05 | 13.80 | 7.68 | 5.23 | 6.15 | 7.86 | 6.65 | 6.72 | 3.62 | 7.10 | 10.55 |
| 9.17 | 8.21 | 4.78 | 2.73 | 6.78 | 9.34 | 11.36 | 9.18 | 7.94 | 13.29 | 5.34 | 5.30 | 6.80 | 7.13 | 5.14 | 4.89 | 3.77 | 6.14 | 8.37 |
| 8.96 | 8.03 | 3.67 | 0.47 | 5.16 | 10.50 | 11.49 | 6.21 | 7.86 | 12.53 | 3.22 | 5.84 | 7.70 | 6.48 | 4.06 | 3.12 | 3.83 | 5.52 | 6.08 |
| 8.65 | 8.00 | 2.70 | -0.94 | 3.84 | 12.26 | 11.68 | 3.26 | 7.87 | 11.64 | 1.30 | 6.44 | 8.47 | 5.78 | 3.23 | 1.72 | 3.49 | 5.05 | 4.25 |
| 8.35 | 8.40 | 2.13 | -1.67 | 2.89 | 13.95 | 11.70 | 0.65 | 7.90 | 10.65 | -0.41 | 6.52 | 8.73 | 4.98 | 2.37 | 0.67 | 2.61 | 4.74 | 3.06 |
| 7.87 | 9.12 | 1.83 | -2.04 | 2.15 | 14.96 | 11.34 | -1.41 | 7.88 | 9.49 | -1.84 | 5.97 | 8.44 | 4.11 | 1.38 | -0.14 | 1.64 | 4.95 | 2.32 |
| 7.31 | 9.84 | 1.68 | -2.07 | 1.51 | 15.28 | 10.42 | -2.85 | 7.94 | 8.28 | -2.83 | 5.36 | 8.18 | 3.40 | 0.48 | -0.66 | 1.39 | 6.04 | 1.80 |
| 7.18 | 10.33 | 1.43 | -1.83 | 1.27 | 15.21 | 9.34 | -3.54 | 8.11 | 7.22 | -3.24 | 5.56 | 8.47 | 2.98 | -0.06 | -0.79 | 2.15 | 7.71 | 1.26 |
| 7.80 | 10.79 | 1.19 | -1.52 | 1.65 | 15.21 | 8.76 | -3.70 | 8.45 | 6.55 | -3.16 | 7.41 | 9.40 | 2.89 | -0.04 | -0.60 | 3.60 | 8.98 | 0.77 |
| 9.00 | 11.44 | 1.09 | -1.26 | 2.37 | 15.57 | 9.44 | -3.69 | 9.11 | 6.49 | -2.77 | 10.57 | 10.58 | 3.22 | 0.52 | -0.44 | 5.26 | 9.11 | 0.40 |
| 10.38 | 11.98 | 1.28 | -1.04 | 2.68 | 16.28 | 11.36 | -3.64 | 10.05 | 7.08 | -2.34 | 13.57 | 11.59 | 4.08 | 1.47 | -0.77 | 6.88 | 8.20 | 0.18 |
| 11.69 | 12.18 | 1.79 | -0.79 | 2.12 | 17.15 | 13.66 | -3.41 | 11.10 | 8.06 | -1.85 | 14.90 | 12.38 | 5.61 | 2.49 | -1.55 | 8.37 | 7.37 | 0.32 |
| 12.78 | 11.87 | 2.42 | -0.45 | 1.05 | 18.00 | 15.38 | -2.88 | 11.89 | 8.85 | -1.24 | 14.32 | 13.31 | 7.35 | 3.11 | -2.71 | 9.38 | 7.95 | 0.83 |
| 13.29 | 11.14 | 2.90 | -0.16 | -0.20 | 18.64 | 15.53 | -2.35 | 12.15 | 8.90 | -0.63 | 12.81 | 14.24 | 8.63 | 2.90 | -4.28 | 9.69 | 9.90 | 1.26 |
| 13.04 | 10.34 | 3.13 | -0.01 | -1.27 | 18.91 | 14.03 | -1.92 | 11.69 | 8.14 | -0.17 | 11.86 | 14.76 | 8.95 | 1.91 | -6.18 | 9.49 | 11.76 | 0.98 |
| 12.41 | 9.82 | 3.30 | 0.19 | -1.92 | 18.75 | 11.89 | -1.41 | 10.75 | 7.03 | 0.22 | 12.22 | 14.34 | 8.48 | 0.81 | -7.71 | 9.30 | 12.36 | 0.09 |
| 11.79 | 9.76 | 3.69 | 0.79 | -1.51 | 18.20 | 10.22 | -0.20 | 9.69 | 6.36 | 0.84 | 13.20 | 13.25 | 7.86 | 0.36 | -8.06 | 9.32 | 11.65 | -0.47 |
| 11.01 | 10.13 | 4.61 | 1.83 | 0.25 | 17.18 | 10.04 | 1.92 | 9.07 | 6.55 | 1.92 | 13.69 | 12.16 | 7.64 | 1.02 | -6.99 | 9.11 | 10.45 | 0.01 |
| 9.85 | 10.92 | 6.05 | 3.09 | 2.84 | 15.95 | 11.44 | 4.25 | 9.19 | 7.57 | 3.28 | 13.07 | 11.70 | 7.92 | 2.44 | -5.04 | 8.46 | 9.54 | 1.36 |
| 8.81 | 12.00 | 7.87 | 4.16 | 5.25 | 15.16 | 13.27 | 5.93 | 10.03 | 8.92 | 4.45 | 12.12 | 12.16 | 8.49 | 3.98 | -2.94 | 7.78 | 9.13 | 2.62 |
| 8.61 | 13.18 | 9.73 | 4.90 | 6.92 | 15.29 | 14.71 | 6.79 | 11.36 | 10.17 | 5.21 | 11.60 | 13.20 | 9.03 | 5.12 | -1.00 | 7.47 | 9.22 | 3.29 |
| 9.06 | 13.98 | 11.20 | 5.58 | 7.71 | 15.86 | 15.59 | 7.26 | 12.67 | 11.05 | 5.63 | 11.27 | 14.23 | 9.36 | 5.82 | 0.63 | 7.56 | 9.89 | 3.52 |
| 9.36 | 13.95 | 11.94 | 6.34 | 8.02 | 16.25 | 16.35 | 7.91 | 13.36 | 11.46 | 5.86 | 10.66 | 14.63 | 9.42 | 6.18 | 1.79 | 7.78 | 10.85 | 3.69 |
| 9.12 | 12.94 | 11.86 | 6.92 | 7.91 | 16.27 | 17.05 | 8.58 | 13.19 | 11.47 | 6.01 | 9.84 | 14.24 | 9.27 | 6.23 | 2.21 | 7.92 | 11.78 | 3.91 |
| 8.63 | 11.07 | 11.31 | 6.95 | 7.12 | 15.94 | 17.40 | 8.87 | 12.24 | 11.15 | 5.86 | 8.95 | 13.23 | 9.18 | 6.05 | 1.92 | 8.02 | 12.44 | 4.02 |
| 8.06 | 9.04 | 10.69 | 6.44 | 5.92 | 15.33 | 17.24 | 8.60 | 10.80 | 10.59 | 5.71 | 7.87 | 12.04 | 9.31 | 5.96 | 1.36 | 7.94 | 13.06 | 4.14 |
| 7.37 | 7.54 | 10.31 | 5.89 | 4.82 | 14.46 | 16.95 | 8.08 | 9.29 | 10.06 | 5.68 | 6.75 | 10.96 | 9.78 | 6.28 | 1.26 | 7.60 | 13.53 | 4.65 |
| 6.75 | 6.51 | 10.17 | 5.63 | 4.47 | 13.55 | 16.54 | 7.65 | 7.94 | 9.54 | 5.76 | 6.23 | 10.19 | 10.35 | 7.05 | 1.97 | 7.25 | 13.58 | 5.59 |
| 6.25 | 5.47 | 9.96 | 5.53 | 4.80 | 12.72 | 15.11 | 7.35 | 6.67 | 8.91 | 5.63 | 6.45 | 9.64 | 10.61 | 7.74 | 3.14 | 6.93 | 12.98 | 6.48 |
| 5.61 | 4.16 | 9.30 | 5.21 | 5.14 | 11.88 | 12.20 | 7.05 | 5.32 | 7.93 | 5.06 | 6.73 | 9.03 | 10.13 | 7.77 | 4.02 | 6.48 | 12.00 | 6.75 |
| 4.63 | 3.07 | 8.22 | 4.47 | 5.02 | 10.76 | 9.19 | 6.55 | 4.13 | 6.75 | 4.09 | 6.28 | 8.57 | 9.02 | 7.05 | 4.03 | 6.05 | 11.20 | 6.24 |
| 3.62 | 2.85 | 7.10 | 3.35 | 4.55 | 9.53 | 8.19 | 5.84 | 3.42 | 5.60 | 3.13 | 5.39 | 8.46 | 7.92 | 6.02 | 3.46 | 5.95 | 11.11 | 5.38 |
| 2.97 | 3.41 | 6.12 | 1.94 | 3.86 | 8.45 | 9.63 | 4.96 | 3.34 | 4.67 | 2.47 | 4.81 | 8.78 | 7.15 | 5.07 | 2.85 | 6.29 | 11.64 | 4.61 |
| 2.87 | 4.15 | 5.19 | 0.47 | 3.11 | 7.83 | 11.12 | 3.80 | 3.68 | 3.99 | 2.17 | 5.27 | 9.19 | 6.66 | 4.25 | 2.41 | 6.76 | 12.05 | 4.13 |
| 3.40 | 4.77 | 4.11 | -0.78 | 2.33 | 7.77 | 10.45 | 2.58 | 3.98 | 3.49 | 2.07 | 6.67 | 9.17 | 6.06 | 3.44 | 2.02 | 7.23 | 11.87 | 4.04 |
| 4.55 | 5.05 | 3.00 | -1.61 | 1.71 | 8.05 | 7.92 | 1.68 | 3.93 | 3.06 | 1.82 | 8.16 | 8.66 | 5.26 | 2.52 | 1.66 | 7.48 | 10.88 | 4.07 |
| 5.65 | 4.70 | 2.19 | -1.78 | 1.41 | 8.05 | 5.51 | 1.48 | 3.58 | 2.67 | 1.40 | 8.64 | 7.82 | 4.46 | 1.58 | 1.46 | 7.47 | 9.56 | 4.06 |
| 5.68 | 3.99 | 1.84 | -1.46 | 1.49 | 7.13 | 4.32 | 1.87 | 3.05 | 2.33 | 0.67 | 7.91 | 6.94 | 3.95 | 0.82 | 1.77 | 7.27 | 8.61 | 3.93 |
| 4.60 | 3.63 | 1.94 | -0.93 | 1.78 | 5.49 | 4.01 | 2.46 | 2.71 | 2.18 | -0.19 | 6.98 | 6.15 | 3.75 | 0.45 | 2.59 | 7.19 | 8.38 | 3.95 |
| 3.51 | 4.14 | 2.21 | -0.56 | 1.97 | 4.04 | 4.16 | 2.77 | 2.77 | 2.25 | -1.03 | 6.79 | 5.34 | 3.58 | 0.43 | 3.55 | 7.68 | 9.25 | 4.39 |
| 3.54 | 5.10 | 2.41 | -0.90 | 1.80 | 3.37 | 4.49 | 2.35 | 3.09 | 2.34 | -1.99 | 7.16 | 4.65 | 2.99 | 0.47 | 4.21 | 8.84 | 10.49 | 5.08 |
| 4.74 | 5.36 | 2.43 | -1.92 | 1.34 | 3.46 | 4.73 | 1.32 | 3.48 | 2.16 | -3.16 | 7.67 | 4.55 | 2.00 | 0.38 | 4.48 | 10.06 | 10.75 | 5.53 |
| 6.19 | 4.49 | 2.42 | -2.98 | 1.06 | 3.96 | 4.76 | 0.31 | 3.75 | 1.75 | -4.30 | 8.18 | 4.93 | 0.92 | 0.19 | 4.68 | 10.69 | 9.48 | 5.55 |
| 7.20 | 3.21 | 2.67 | -3.33 | 1.30 | 4.56 | 4.60 | 0.02 | 3.91 | 1.25 | -5.13 | 8.69 | 5.24 | 0.22 | -0.01 | 5.06 | 10.40 | 7.66 | 5.18 |
| 7.58 | 2.49 | 3.24 | -3.00 | 1.72 | 5.06 | 4.33 | 0.62 | 4.04 | 0.96 | -5.53 | 9.03 | 5.12 | -0.15 | -0.34 | 5.35 | 9.47 | 6.56 | 4.48 |
| 7.52 | 2.65 | 3.96 | -2.51 | 1.76 | 5.34 | 3.88 | 1.50 | 4.19 | 0.83 | -5.74 | 9.00 | 4.79 | -0.42 | -0.94 | 5.09 | 8.57 | 6.38 | 3.59 |
| 7.25 | 3.33 | 4.52 | -2.11 | 1.38 | 5.41 | 3.51 | 2.17 | 4.61 | 0.84 | -5.92 | 8.50 | 5.08 | -0.54 | -1.52 | 4.24 | 8.34 | 6.23 | 2.77 |
| 7.16 | 4.25 | 4.89 | -1.61 | 1.14 | 5.60 | 3.74 | 2.66 | 5.25 | 1.10 | -5.80 | 7.84 | 6.19 | -0.30 | -1.67 | 3.44 | 8.86 | 5.77 | 2.38 |
| 7.47 | 5.27 | 5.33 | -0.80 | 1.44 | 6.08 | 4.55 | 3.32 | 6.01 | 1.76 | -5.06 | 7.12 | 7.29 | 0.58 | -1.11 | 3.24 | 9.92 | 5.26 | 2.53 |
| 7.99 | 6.29 | 5.91 | 0.21 | 2.25 | 6.52 | 5.31 | 4.20 | 6.75 | 2.66 | -3.66 | 6.71 | 7.58 | 1.88 | -0.09 | 3.68 | 10.97 | 5.15 | 2.90 |
| 8.10 | 7.14 | 6.56 | 0.97 | 3.29 | 6.56 | 5.53 | 5.34 | 7.27 | 3.57 | -2.01 | 6.70 | 7.14 | 3.19 | 0.96 | 4.27 | 11.54 | 5.70 | 3.28 |
| 7.49 | 7.84 | 7.22 | 1.37 | 4.55 | 6.31 | 5.78 | 6.91 | 7.62 | 4.31 | -0.26 | 6.99 | 6.95 | 4.39 | 2.03 | 4.83 | 11.71 | 7.02 | 3.80 |
| 6.71 | 8.51 | 8.19 | 1.63 | 6.08 | 6.31 | 6.95 | 9.18 | 8.01 | 5.08 | 1.63 | 7.12 | 7.76 | 5.59 | 3.21 | 5.55 | 11.79 | 8.85 | 4.57 |
| 6.46 | 9.22 | 9.74 | 2.38 | 7.87 | 6.81 | 9.42 | 11.89 | 8.60 | 6.15 | 3.93 | 7.03 | 9.37 | 7.00 | 4.66 | 6.84 | 11.93 | 10.54 | 5.79 |
| 7.06 | 9.81 | 11.72 | 4.00 | 9.54 | 7.52 | 12.37 | 14.31 | 9.45 | 7.62 | 6.49 | 6.88 | 10.80 | 8.57 | 6.40 | 8.63 | 12.11 | 11.39 | 7.36 |
| 8.20 | 10.22 | 13.50 | 6.14 | 10.64 | 8.04 | 14.61 | 15.73 | 10.46 | 9.22 | 8.73 | 7.22 | 11.50 | 10.16 | 8.26 | 10.28 | 12.37 | 11.25 | 8.70 |
| 9.29 | 10.62 | 14.77 | 8.00 | 10.88 | 8.45 | 15.51 | 16.10 | 11.50 | 10.64 | 10.10 | 8.21 | 11.63 | 11.37 | 9.87 | 11.13 | 12.78 | 10.51 | 9.38 |
| 9.79 | 11.39 | 15.48 | 9.09 | 10.44 | 9.05 | 15.37 | 15.94 | 12.41 | 11.64 | 10.51 | 9.22 | 11.52 | 11.96 | 11.00 | 11.05 | 13.23 | 10.05 | 9.31 |
| 9.53 | 12.50 | 15.83 | 9.40 | 9.84 | 9.99 | 14.81 | 15.66 | 12.86 | 12.10 | 10.37 | 9.67 | 11.33 | 11.82 | 11.61 | 10.54 | 13.50 | 10.28 | 8.90 |
| 9.14 | 13.49 | 15.93 | 9.41 | 9.43 | 10.98 | 14.06 | 15.23 | 12.72 | 12.04 | 10.06 | 9.48 | 11.18 | 11.27 | 11.88 | 10.12 | 13.49 | 10.78 | 8.68 |
| 9.59 | 13.82 | 15.83 | 9.28 | 9.17 | 11.70 | 13.45 | 14.42 | 12.26 | 11.67 | 9.87 | 8.92 | 11.23 | 10.81 | 12.21 | 9.90 | 13.42 | 11.07 | 8.76 |
| 11.17 | 13.51 | 15.77 | 8.91 | 8.91 | 12.07 | 13.23 | 13.56 | 12.13 | 11.40 | 9.90 | 8.17 | 11.51 | 10.92 | 12.90 | 9.95 | 13.33 | 10.89 | 9.19 |
| 12.74 | 12.84 | 15.94 | 8.36 | 8.57 | 11.96 | 13.45 | 12.91 | 12.45 | 11.45 | 10.06 | 7.60 | 11.83 | 11.37 | 13.80 | 10.19 | 13.13 | 10.29 | 9.65 |
| 13.07 | 12.23 | 16.21 | 7.72 | 8.32 | 11.54 | 14.27 | 12.69 | 13.11 | 11.78 | 10.37 | 7.66 | 12.16 | 11.75 | 14.59 | 10.61 | 13.04 | 9.30 | 9.93 |
| 12.08 | 11.83 | 16.31 | 7.50 | 8.25 | 10.98 | 15.48 | 12.87 | 13.56 | 12.15 | 10.67 | 8.79 | 12.53 | 11.74 | 14.81 | 11.06 | 13.35 | 7.83 | 10.01 |
| 10.57 | 11.41 | 16.01 | 7.85 | 8.62 | 10.54 | 16.53 | 13.32 | 13.49 | 12.21 | 10.80 | 10.30 | 12.84 | 11.28 | 14.46 | 11.37 | 13.57 | 6.40 | 9.95 |
| 9.46 | 11.09 | 15.32 | 8.50 | 9.50 | 10.37 | 16.76 | 13.93 | 13.03 | 11.90 | 10.64 | 10.95 | 12.64 | 10.43 | 13.62 | 11.78 | 13.00 | 5.70 | 9.84 |
| 9.15 | 10.96 | 14.37 | 8.95 | 10.65 | 10.16 | 15.98 | 14.27 | 12.49 | 11.23 | 10.08 | 10.19 | 11.51 | 9.23 | 12.27 | 12.18 | 11.10 | 5.82 | 9.41 |
| 9.35 | 10.86 | 13.07 | 8.66 | 11.57 | 9.60 | 14.66 | 13.95 | 12.18 | 10.32 | 9.07 | 8.31 | 9.96 | 7.67 | 10.53 | 12.21 | 8.74 | 6.28 | 8.48 |
| 9.50 | 10.76 | 11.49 | 7.59 | 11.78 | 8.58 | 13.16 | 13.05 | 12.03 | 9.34 | 7.74 | 6.07 | 9.09 | 6.01 | 8.66 | 11.64 | 7.36 | 6.82 | 7.47 |
| 8.94 | 10.69 | 9.75 | 6.11 | 11.18 | 7.32 | 11.98 | 12.03 | 11.94 | 8.39 | 6.30 | 4.42 | 9.71 | 4.54 | 7.14 | 10.61 | 7.56 | 7.47 | 7.12 |

Each cell corresponds to a time instant starting from 5 ms to 1000 ms (y-axis) in every scalp electrode site (x-axis) where the EEG signal was recorded. Averaged-voltage values are in microvolts (μV).
